# Updating global green-hydrogen production costs and configurations under future climates

**DOI:** 10.1016/j.xinn.2026.101303

**Published:** 2026-02-05

**Authors:** Haochi Wu, Mingyang Sun, Michael T. Craig

**Affiliations:** 1 School for Control Science and Engineering, Zhejiang University, Hangzhou, Zhejiang 310027, China; 2 School for Environment and Sustainability, University of Michigan, Ann Arbor, MI 48109, USA; 3 Department of Control Science and Systems Engineering, School of Advanced Manufacturing and Robotics, Peking University, Beijing 100871, China; 4 Department of Civil and Environmental Engineering, Stanford University, Stanford, CA 94305, USA; 5 Department of Industrial and Operations Engineering, University of Michigan, Ann Arbor, MI 48109, USA

**Keywords:** green hydrogen, levelized cost of hydrogen, LCOH, climate change impacts, hydrogen-system optimization, renewable-energy systems

## Abstract

Meeting global decarbonization targets requires large-scale, low-carbon hydrogen (H_2_) production around mid-century. A crucial pathway for this production is electrolysis driven by renewables, tying hydrogen production and costs to spatially varying renewable resources. The potential, variability, and complementarity of renewable resources, though, will be affected by climate change. We quantify the impact of climate change on renewable-energy generation for H_2_ production globally. We use an investment and operations optimization model for hydrogen systems to estimate geographically explicit and regionally aggregated levelized cost of hydrogens (LCOHs) under historical and future climates. We find climate change could raise the cost of green-hydrogen production by up to 20% in some global locations, and about 16% of global locations could see LCOH increases or decreases exceeding 5%. Southeast Asia and Europe in particular see LCOH reductions due to climate change, while North America sees LCOH increase. Most locations, though, see modest impacts of climate change on hydrogen costs. We also find modest cost consequences from climate change for locations with active hydrogen development. Our results highlight the need for proactive investment strategies to accommodate the climatic variations affecting renewable hydrogen production, especially in countries with stricter H_2_ power-grid import limits and with firm H_2_ demand for industrial processes.

## Public summary

• Climate change has a modest impact on green-hydrogen costs across most regions.
• Costs at high-potential locations change by less than 10% due to climate.
• Future climate change increases hydrogen storage requirements by ∼20%.
• Weather variability affects hydrogen costs more significantly than climate change.

## Graphical abstract

**Figure:**
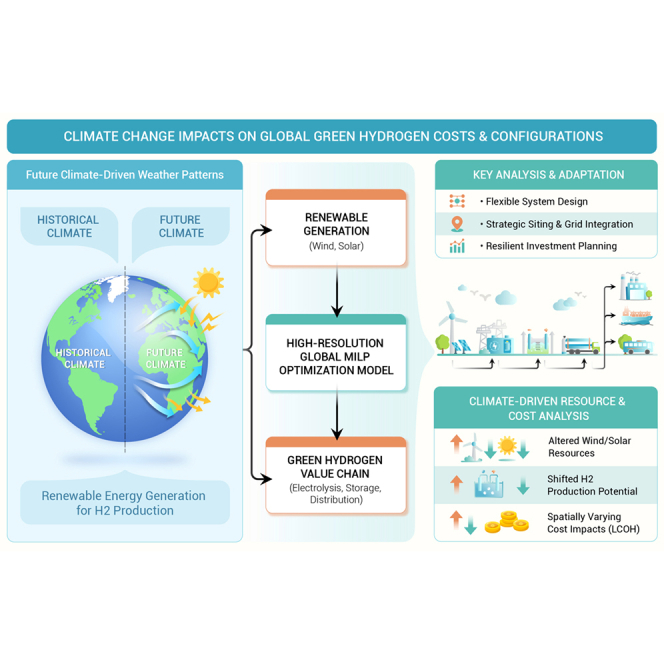

## Introduction

Production and use of low-carbon hydrogen (H_2_) is critical for decarbonizing the global economy.^1^^,^^2^^,^^3^ Most decarbonization scenarios have hydrogen and its derivatives playing a key role in decarbonization of transport, food, industrial, and power sectors in nearly all global regions.^4^^,^^5^ Hydrogen deployment, especially with new applications in these sectors, typically begins around 2035 and reaches widespread use around 2050.^6^

For hydrogen to contribute to aggressive decarbonization targets, low emissions intensity in hydrogen production is required.^1^^,^^2^^,^^4^ Among the pathways for creating low-carbon hydrogen, production via electrolysis powered by renewables such as wind and solar electricity is the most promising.^7^ This pathway for hydrogen production can be completed through dedicated wind and solar power for electrolysis or through grid-connected electrolyzers on grids where wind and solar power account for most or all electricity generation. In either case, hydrogen production costs and carbon intensity will vary across space and time based on investment needs and operations driven by variability in wind and solar resources.^8^^,^^9^

Several recent studies have quantified the cost of hydrogen production via wind- and solar-driven electrolysis on regional and global scales for various sectors.^8^^,^^9^^,^^10^^,^^11^^,^^12^^,^^13^^,^^14^ These studies generally indicate large differences across space in hydrogen costs and optimal investments in hydrogen production systems. For instance, Terlouw et al.^13^ find costs range from 1 to 8 Euros per kg of hydrogen production. Two key factors differentiate the methodologies of these studies. First, studies differ with respect to whether they consider hydrogen production using grid-connected or dedicated wind and solar power. In both cases, studies consider geographically explicit (or latitude- and longitude-specific) energy inputs. Regional analyses in these studies do not blend wind and solar across locations, as would occur in grid-connected systems, but instead use wind and solar resources from the same location, likely underestimating the value of regional aggregation. Second, studies differ in the degree to which they consider the system dynamics of hydrogen production and consumption. Some studies optimize investment in all components of a hydrogen system, including wind, solar, electrolysis, and storage capacity, while other studies use cruder metrics (e.g., levelized costs) to approximate system designs. Studies also differ in the degree to which they analyze hydrogen demand, ranging from satisfying hourly to annual demands. Studies that optimize system design while capturing important hourly supply and demand dynamics generally analyze limited locations,^15^^,^^16^^,^^17^ while studies that conduct a rougher analysis of system design have been applied to a larger geographic area, in some cases global^8^^,^^11^^,^^13^^,^^14^ (Table S1). Prior work indicates ignoring system optimization can underestimate investment needs (e.g. in renewables and storage) and therefore underestimate hydrogen costs, underscoring the importance of detailed system optimization.^17^

Another recent body of literature has examined the effect of climate change on wind and solar resource variability and complementarity.^18^^,^^19^^,^^20^^,^^21^^,^^22^ These studies generally indicate mild effects on wind and solar potential, temporal variability, and complementarity up to 15% of changes in most regions. Renewable-energy-based hydrogen deployment is not expected to scale up until roughly 2035, and hydrogen-related energy infrastructure is expected to have multi-decade lifespans. For both reasons, hydrogen systems will not operate under present or historical weather conditions but, rather, under future weather conditions affected by climate change. However, no studies have examined hydrogen-system design and operation under internal climate variability at a global level or under future climate variability driven by climate change.

In this study, our research question is: how will climate change affect the economics of hydrogen production through wind- and solar-driven electrolysis on a global basis? In answering this question, we make two contributions to the literature. First, we analyze the impact of internal variability and climate change on optimal hydrogen-system design and costs on a global scale. In so doing, we model hydrogen costs and system designs under the weather they will experience, providing a more accurate quantification of hydrogen-system designs and costs. Second, we provide a global analysis of hydrogen-system design using a detailed system-optimization model applied to geographically explicit and to regional system designs. In so doing, we provide a unique perspective into global hydrogen-system designs and costs.

We quantify the economics of hydrogen production by calculating levelized costs of hydrogen (LCOHs) under historical and future climates. The LCOH quantifies the average cost per unit of produced hydrogen over the hydrogen system’s lifetime. We estimate LCOHs using a mixed-integer linear program (MILP) that minimizes fixed plus variable costs by optimizing hydrogen-system investment decisions given hourly operational details. Investments and operations are optimized for solar photovoltaics (PV), wind turbines, electrolyzers, batteries, compressors, hydrogen storage tanks, and grid power purchases. We derive location-specific hourly wind and solar resources using output from eight Coupled Model Intercomparison Project 6 (CMIP6) global climate models (GCMs) for a historical (2000–2010) and future (2065–2075) period. Our future climate data are obtained for a Shared Socioeconomic Pathway 585 (SSP5-8.5) pathway. We use an ensemble of eight state-of-the-art CMIP6 GCMs with different equilibrium climate sensitivity to enhance the robustness of our result. We run our optimization model for geographically explicit locations at 1° × 1° resolution and for aggregated regions, thereby estimating LCOH costs for off- and on-grid hydrogen systems.

## Materials and methods

### Hydrogen-system investment and operation model

**Figure 1:**
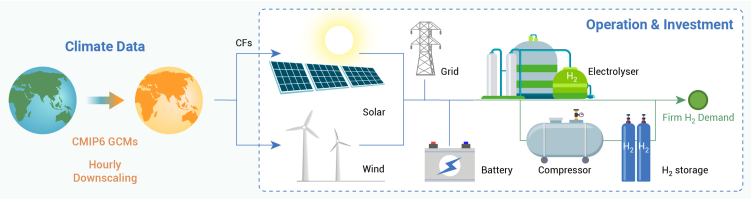
Schematic for the overall framework of hydrogen-system analysis under climate changes

We construct an MILP to optimize hydrogen-system investment decisions given hourly operational details,^12^^,^^13^ as summarized in Figures 1 and S1. We define “hydrogen system” to include six components: solar PV, wind turbines, electrolyzer, batteries, compressors, and hydrogen storage tanks. Thus, our MILP optimizes capacity investment in and operations of each of these components. Like prior literature, we also allow the hydrogen system to purchase electricity from the bulk power system, which incurs purchase costs.

The MILP minimizes total costs *z*_cost_, as follows:

(Equation 1) $$\min\;z_{\text{cost}}=\sum_{k\in K}I_{k}+\sum_{k\in K}v_{k}I_{k}+\sum_{l=1}^{L}\sum_{t=0}^{T}p_{E}\left(M_{E,t}\right)\text{,}$$

where total costs include capital costs (*I*_*k*_), operation and maintenance (O&M) costs (*v*_*k*_*I*_*k*_), and costs of purchasing grid power (*p*_E_(*M*_E,*t*_)); where *l* indexes years in the lifetime of the hydrogen system (25 years), *t* indexes hours in a year, and *k* indexes technology; and where *v*_*k*_ is the annual maintenance fraction of the installation cost, *p*_E_ is the price of grid electricity for industrial users, and *M*_E,*t*_ indicates the quantity of electricity imported from the grid.

Model constraints ensure compliance with grid power import limits, balance between energy supply and demand, storage operations, and technological limitations of each component in the system, e.g., time-varying wind and solar capacity factors, similar to other energy systems studies.^23^ Constraints capture operational characteristics and limitations for the hydrogen system as a whole, as well as for individual technologies in the system. Additional details on cost calculation can be found in Note S1.

Given a set of investment decisions and operations, we compute the LCOH, which quantifies the average cost per unit of hydrogen produced, as

(Equation 2) $$\text{LCOH}=\frac{z_{\text{cost}}}{\sum_{l=1}^{L}\sum_{t=0}^{T}D_{H2,t}}\text{.}$$

We assume the required total amount of hydrogen produced $\sum_{l=1}^{L}\sum_{t=0}^{T}D_{H2,t}$, where *D*_H2,*t*_ is the hourly hydrogen output of the modeled hydrogen systems, remains consistent regardless of the chosen assumptions regarding input parameters. This allows for a meaningful comparison of LCOH across diverse scenarios.^12^

We run our above investment and operational model in two modes, allowing us to separate the effects of climate change from internal variability. In the first mode, we run our model for each hour in a year on a year-by-year basis for 10 years, inputting hourly wind and solar capacity factors for a given year. We then average annual results to obtain the optimal system configuration for a specific climatology. This mode yields our main set of results.

In the second mode, we run our model over all 10 years to optimize H_2_ investment and operations across all years, thereby capturing the effect of internal variability and allowing us to compare this effect against the effect of climate change (Figure 7). Figure S8 compares results in the future climate between these two modes. In optimizing over 10 years, our model constraint requires total hydrogen production to meet or exceed the yearly demand over the entire investment horizon.

(Equation 3) $$D_{H_{2}}\left(t,y\right)\geq \text{Hourly}\;H_{2}\;\text{demand}\left(t,y\right)\;\forall t\in \left\{1,\ldots ,T\right\},\forall y\in \left\{1,\ldots ,Y\right\}$$

Here, *Y* represents the number of years in the investment horizon, *T* is the number of hours in a year (8,760), $D_{H_{2}}\left(t,y\right)$ is the hydrogen demand at time *t* in year *y*, and Hourly H_2_demand(*t*,*y*) is the hourly hydrogen demand at time *t* for year *y*. A similar constraint is included in the first mode in which we run our model, but Y in that case is set equal to 1, and the model is run separately for each year. In the multi-year mode, Y is set equal to 10 years.

### Simplified LCOE-based cost calculation

**Figure 2:**
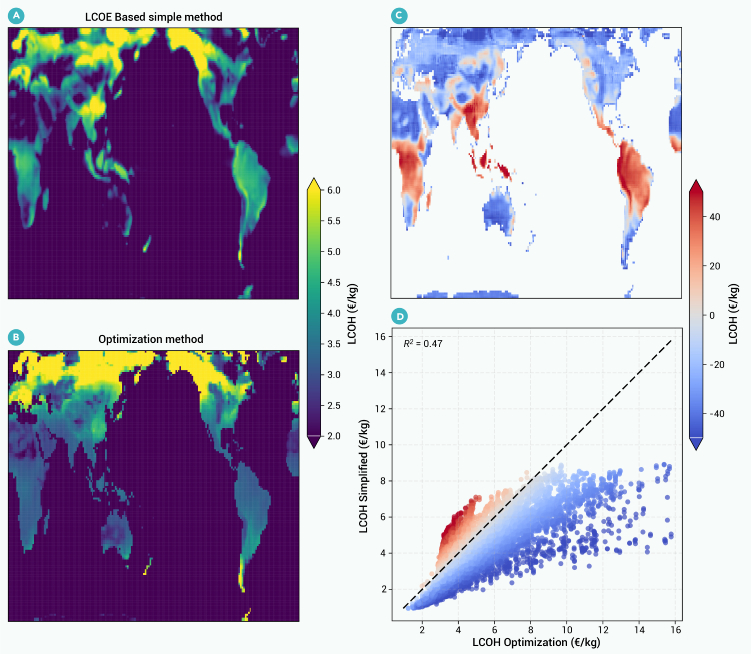
Simplified versus optimization-based LCOH estimates under historical climate (A and B) LCOH using a simple LCOE-based model using the same design assumptions from de Kleijne et al.^8^ (A) and a dynamic system-optimization model (B). (C and D) (C) The change in LCOH (in %) from the dynamic optimization method (regarded as ground truth) to simplified LCOE method, while (D) compares the two sets of LCOH estimates (with a *y* = *x* line superimposed) with dots representing global locations; dot color indicates the LCOH error corresponding to the map in (C).

Prior literature examining hydrogen investments and operations at a global level has used a simplified approach, like Levelized Cost of Energy (LCOE) based method. to optimizing the hydrogen system.^8^^,^^14^ We compare our detailed system optimization against these simplified approaches by running our analysis using a simplified model like those used in prior research. The results of this simplified approach are only included in Figure 2; the remaining figures include results from our detailed system-optimization model from the prior section. The simplified model ignores operational constraints that are included in our detailed optimization model. As a result, our simplified model, like simplified models in prior research, must make assumptions about investment needs that would otherwise be dictated by operational constraints.

With our simplified model, we calculate the LCOH using Equation 4:

(Equation 4) $$\text{LCOH}=\frac{I_{\text{total}}}{\sum_{l=1}^{L}A_{H2,l}}$$

where *I*_total_ is total annualized investment and operational costs ($), including capital and operational expenses for solar PV, wind turbines, and electrolyzers; and *A*_H2,*l*_ is the annual hydrogen production (kg/year) for single year *l* calculated based on renewable-energy generation. This simplified model only captures investment costs of wind turbines, solar PV, and electrolyzers (similar to prior research) because it does not model operations of the hydrogen system, in which storage and other costs would be relevant that are included in our full optimization model detailed in the prior section.

The total cost *I*_total_ is calculated in Equation 5:

(Equation 5) $$I_{\text{total}}=\underset{\text{Solar}\;\text{PV}}{\underset{︸}{P_{\text{PV}}\cdot \left(c_{PV}+OM_{PV}\right)}}+\underset{\text{Wind}}{\underset{︸}{P_{\text{WT}}\cdot \left(c_{WT}+OM_{WT}\right)}}+\underset{\text{Electrolyzer}}{\underset{︸}{P_{\text{EL}}\cdot \left(c_{EL}+OM_{EL}\right)}}\text{,}$$

where *P*_PV_ and *P*_WT_ are the installed capacities (in MW) of the solar PV and wind turbine technologies, respectively; *c*_*PV*_ and *c*_*WT*_ are the capital costs per MW for solar PV and wind turbines, respectively; *OM*_*PV*_ and *OM*_*WT*_ represent the operations and maintenance costs per MW for solar PV and wind turbines, respectively; *P*_EL_ is the installed capacity of the electrolyzer; and *c*_*EL*_ and *OM*_*EL*_ denote the capital and operations and maintenance costs per MW for the electrolyzer, respectively. Like prior research, we assume a capacity sizing ratio between solar PV, wind, and electrolyzer; in our case, we assume a 1:1:1 ratio, such that *P*_WT_ equals *P*_PV_ equals *P*_EL_.

The equation for annual hydrogen production output *A*_H2_ is in Equation 6:

(Equation 6) $$A_{H2}=E_{RE}\cdot R_{el/re}\cdot \eta_{EL}\text{,}$$

where *E*_*RE*_ is the total renewable energy generated annually and fed into the electrolyzer; *R*_*el*/*re*_ is the ratio of electrolyzer capacity to renewable-energy capacity, representing the oversizing ratio; and *η*_*EL*_ is the efficiency of the electrolyzer in converting renewable electricity into hydrogen.

Finally, the annual renewable-energy generation *E*_*RE*_ is calculated as

(Equation 7) $$E_{RE}=8,760\cdot \left(P_{WT}\cdot CF_{WT}^{avg}+P_{PV}\cdot CF_{PV}^{avg}\right)\text{,}$$

where $CF_{WT}^{avg}$ and $CF_{PV}^{avg}$ are the average annual capacity factors for wind turbines and solar PV, respectively. The factor 8,760 represents the total number of hours in a year, used to convert capacity into annual energy generation.

In this simplified model, unlike our optimization model described in the prior section, for global locations, the heterogeneity and non-linear relationship between LCOE and LCOH in different regions, as observed in Terlouw et al.,^13^ cannot be considered.

### Renewable-energy production under historical and future climates

To quantify the effect of climate change on hydrogen-system configurations and costs, we ran our analytical framework described above under a historical and future climate. Specifically, we selected eight CMIP6 GCMs, similar to prior studies,^24^^,^^25^ then we obtained historical and future climate data for the periods 2000–2010 and 2065–2075, respectively. The future climate data were obtained for the SSP585 scenario, which is a scenario that yields additional radiative forcing of 8.5 *W*/*m*^2^ by the end of the century. We selected the SSP585 scenario, which is a high-warming scenario, to estimate an upper bound of impacts from climate change. As shown in our results, even this upper bound yields modest impacts.

We used 10 years to represent the historical (2000–2010) and future (2065–2075) climatology.^19^ These 10 years capture internal variability in a historical and future climate while maintaining computational tractability, as we ran our system optimization on each grid cell across the globe. The period 2065–2075 captures a period in the middle of the expected lifetime of H_2_ systems deployed in 2050, assuming a near 30-year lifetime. In selecting our eight GCMs, we sampled low to high equilibrium climate sensitivity (ECS) values, but seven of our eight models represented medium or high ECS (Figure S14). We note our modest results despite potentially over-representing medium and high ECSs. In using eight GCMs covering a range of ECS values and analyzing 10 years of climatology, we captured two important sources of climate-related uncertainty in our analysis, specifically model uncertainty and internal variability, increasing confidence in our results.

From each GCM for each period analyzed, we obtained three-hourly climate model outputs for near-surface air temperature, specific humidity, surface pressure, eastward and northward near-surface wind, and surface downwelling shortwave radiation. Linear interpolation was applied to downscale temperature, humidity, wind components, and pressure from three-hourly to hourly data, similar to existing studies.^26^ The hourly solar-elevation angle was also used to downscale solar radiation data.^27^ With these meteorological variables, we calculated location-specific hourly wind and solar capacity factors using commonly used physical relationships (see Note S2 for detailed description).

#### Geographically explicit versus regionally aggregated results

We present our results at two spatial resolutions: geographically explicit and regionally aggregated. Geographically explicit results optimize hydrogen systems at each global location. That is, wind and solar investments available to the model have time series of capacity factors specific to the analyzed location. This geographically explicit analysis is similar to past work.^28^

In contrast, our regionally aggregated results assume grid-connected electrolysis deployment, so optimized wind and solar investments represent investments available within the region’s boundaries. We provide results for 359 regions. Most of these regions correspond to country-level administrative boundaries. However, for specific countries with large land areas, we use secondary administrative units for the division (e.g., states or provinces). Countries for which we use secondary administrative units are the United States, China, Brazil, Australia, Russia, and Canada.

For each region, we calculate a single regionally aggregated solar and wind capacity factor time series from our location-specific timeseries (see renewable-energy production under historical and future climates). Specifically, for each administrative boundary, we select the locations based on the top 20% percentile of annual mean solar and wind capacity factor, respectively. For each hour, we then average the capacity factors of all selected sites to generate a single timeseries for wind and a single timeseries for solar, in which the model can invest. This regional aggregation reduces temporal variability of renewables through complementary siting of wind and solar facilities, allowing for investment in a more stable and reliable renewable electricity supply for hydrogen production (see Note S3 for detailed description and equations). The illustration of locations selected to aggregate solar and wind resources is shown in Figure S12.

## Results

### Comparing geographically explicit hydrogen costs using simple and dynamic investment models

Our first major contribution is to provide a global analysis of hydrogen-system design using a detailed system-optimization model applied to geographically explicit designs. Our dynamic system-optimization model is a MILP designed to co-optimize investment in and hourly operation of a complete green-hydrogen production system. The model determines the least-cost capacity investment of solar PV, wind turbines, electrolyzers, batteries, hydrogen compressors, and storage tanks given hourly constraints on their operation over 1 year (or 8,760 h) or multiple years to meet a firm hydrogen demand. The model’s objective function minimizes total lifetime system costs, including capital costs, ongoing operations and maintenance costs, and costs for purchasing electricity from the bulk power system. The model includes physical and operational constraints for each technology, including hourly wind and solar generation constraints. This integrated approach ensures that the resulting LCOH fully accounts for the system-level costs required to manage renewable variability, a crucial factor that simpler models often overlook (see the section hydrogen-system investment and operation model and Note S1 for more details).

Figure 2 compares the LCOH derived from a dynamic system-optimization model (section hydrogen-system investment and operation model) versus from a simple LCOE-based model (section simplified LCOE-based cost calculation). LCOH values are similar in many parts of the globe using the two methods, but a simple LCOE-based method can over- or underestimate the LCOH by up to 40% in certain parts of the globe (e.g. Southeast Asia, Mexico, South America, and northern Africa). The simple LCOE-based method generally underestimates LCOH costs, and these underestimations are particularly pronounced in locations with high LCOHs (Figure 2D). At locations with low LCOHs, errors between the two methods tend to be smaller. These errors are driven by specific climatic and optimized green-hydrogen-system design factors, as explored in detail through a multivariate regression analysis in Note S4 and Figures S15 and S16.

### Global hydrogen costs and system configurations under climate change

**Figure 3:**
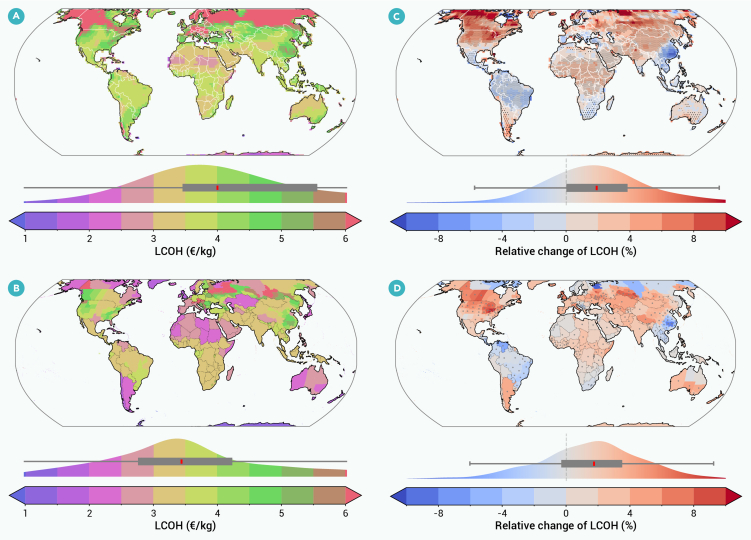
Impact of climate change on global LCOHs (A) Historical LCOH for location-based hydrogen. (B) Historical LCOH for regional hydrogen. (C) Relative change from historical to future climate in LCOH for location-based hydrogen, with dotted area meaning more than six out of eight GCMs model agreement on the direction of changes. (D) Relative change from historical to future climate in LCOH for regional hydrogen, with a gray dot in the region meaning more than six out of eight GCMs model agreement. Above each color bar, a distribution plot and an embedded boxplot summarize the global statistical spread of the mapped values; the box indicates the interquartile range (IQR; 25th to 75th percentiles), with the central red line marking the median. We limit the color bar to 6 €/kg in (A) and (B) and to ±10% in (C) and (D) for clarity; relative changes range from −18 to 26%.

Our second major contribution is to quantify global LCOHs under future weather driven by climate change (i.e., under weather that future hydrogen deployments will operate under). Here and for the rest of our results, we use our detailed system-optimization model to optimize hydrogen-system designs and estimate LCOHs, thereby avoiding errors in LCOH estimates that we documented above using a simplified LCOE model. Figure 3A provides LCOHs for locational hydrogen production under historical conditions, while Figure 3C compares the LCOH under historical (2000–2010) and future (2065–2075) weather under the SSP585 scenario. While climate change could increase the cost of hydrogen production by up to 20%, it has a small effect on LCOHs in many parts of the globe, with most LCOH changes being within ±10%. About 16% of investigated global locations could see LCOH changes exceeding 5%. Climate change tends to increase the LCOH in many parts of the United States, China, and Europe, and it tends to decrease the LCOH in the Iberian Peninsula, Australia, Southeast China, and Southeast Asia. Figure S6A compares the LCOH for each global location under historical and future weather. In general, the effects of climate change are greater at sites with greater LCOH. Sites with LCOHs below 4 €/kg have small changes in LCOH induced by climate change (less than 8%), while sites with LCOHs greater than 12.5 €/kg have larger changes (of roughly 10%–15%). Our result shows broad agreement of H_2_ cost changes among eight selected GCMs at different regions (see Figure S3 for result of eight GCMs separately and Figure S4 for agreement fraction for global locations among all models).

In addition to geographically explicit LCOH and system configuration calculations, we consider regionally aggregated hydrogen costs and configurations. To calculate regionally aggregated values, we use spatially aggregated wind and solar series for selected siting locations within each administrative region, approximating the use of grid-connected wind and solar spread across a region powering grid-connected electrolyzers. Figure 3B provides LCOHs for regional hydrogen production under historical conditions, while the impact of climate change is provided in Figure 3D. Regionally aggregated hydrogen costs are less influenced by climate changes and provide lower LCOHs compared with location-based hydrogen. Figure S6B shows that regional H_2_ costs have generally lower LCOHs with smaller cost changes due to climate change. This result also shows strong model agreement (see Figure S3 for results of eight GCMs separately, and Figure S5 for agreement fraction for global locations among all models).

**Figure 4:**
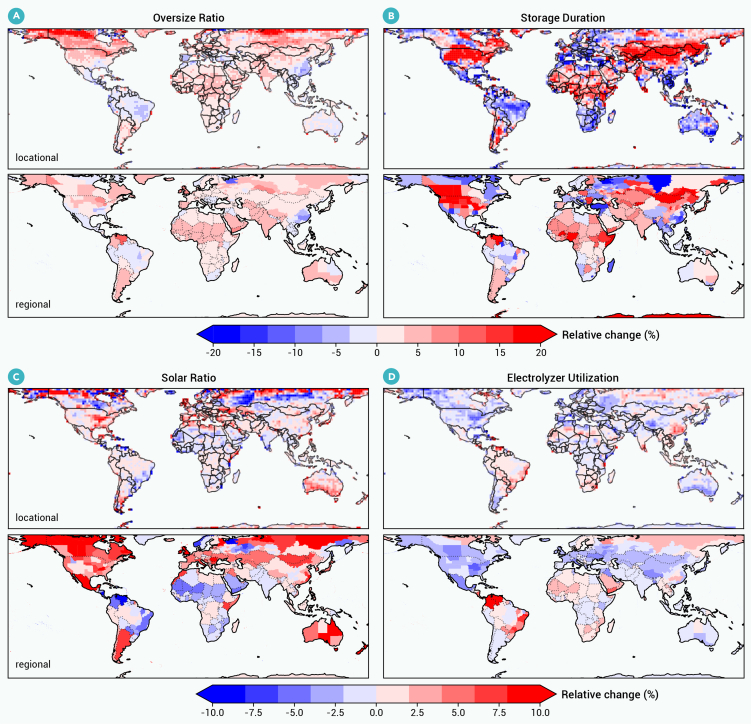
Impact of climate change on hybrid H_2_ system design patterns The top and bottom rows show the relative change in a given system design for location-specific and regional hydrogen, respectively, from historical to future climate. System designs examined are (A) sizing ratio of wind and solar to electrolyzer capacity, (B) hydrogen storage duration, (C) ratio of solar to total wind and solar capacity, and (D) electrolyzer utilization ratio.

**Figure 5:**
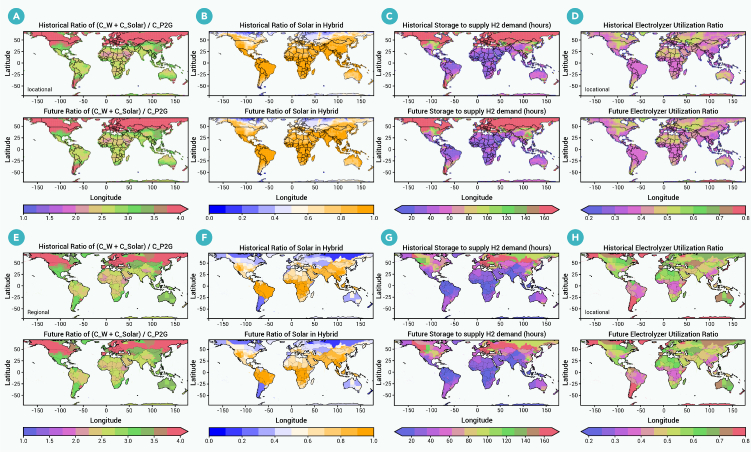
Impact of climate change on hybrid H_2_ system design patterns The first and second rows show historical and future system design patterns locational renewable, while the third and fourth rows are for system with regional renewable, with (A) and (E) over-sizing ratio of renewable to electrolyzer capacity, (B) and (F) ratio of solar in renewable energy, (C) and (G) storage duration, and (D) and (H) electrolyzer utilization ratio.

Climate change affects hydrogen costs through changes to optimal system investments, which are detailed in Figure 4 (see Figure 5 for investment patterns). The largest impact of climate change on investments is in hydrogen storage, which increases across many parts of the globe by up to 20%. Increased investment in storage is needed to offset greater wind and solar variability. The relative investment of solar versus wind is significantly affected by climate change in only a few regions, such as Australia, where solar investment increases by 10%. Electrolyzer investments are largely unresponsive to climate change. The impact of climate change on LCOHs does not demonstrate significant trends against system designs (see Figure S7).

### Climate change influence on global hydrogen supply curve

**Figure 6:**
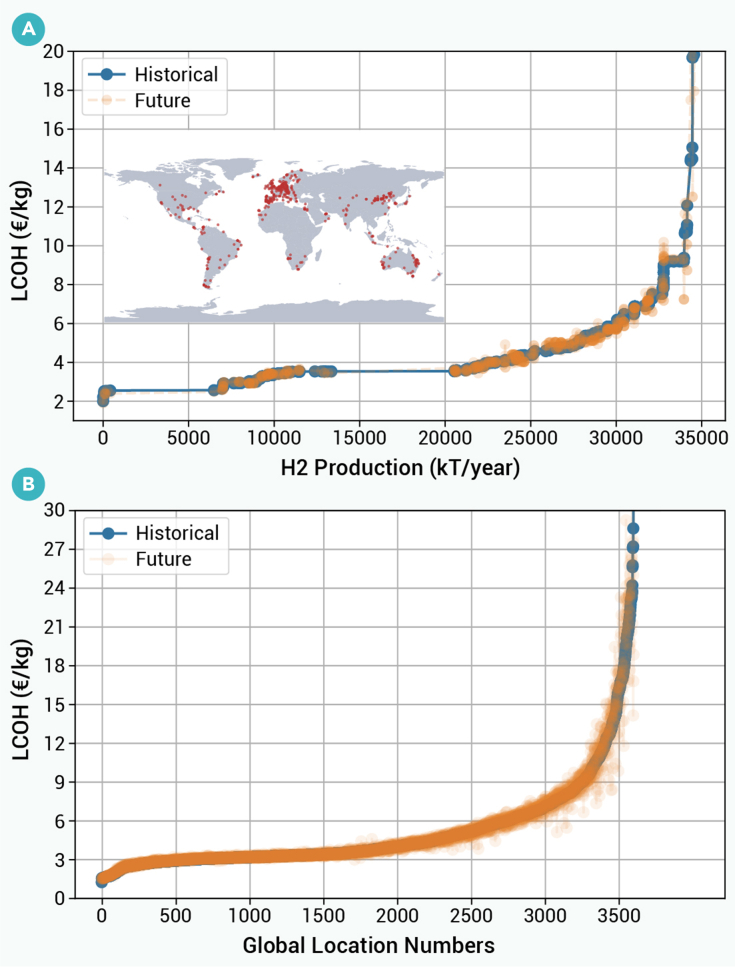
Hydrogen supply curves under historical and future climates Supply curves for (A) announced H_2_ projects and (B) global locations for historical (blue) and future climate (orange) scenarios.

To better understand the effect of climate change on the future supply of hydrogen, we analyze its effect on the supply curve for hydrogen projects under development and for all global locations (Figure 6). Hydrogen projects under development are obtained from the International Energy Agency.^4^ LCOHs of hydrogen projects under development are largely unaffected by climate change, particularly for projects with lower LCOH costs. At LCOH costs below 7.5 €/kg, costs change by less than 8%. A similar phenomenon occurs for the supply curve across all global locations. That is, locations with a low LCOH (e.g., less than 5 €/kg) tend to have negligible cost impacts of climate change, with LCOHs changing by less than 6%. Locations that experience larger impacts of climate change tend to have larger LCOHs.

### Regional investigation of on-site renewable H_2_

**Figure 7:**
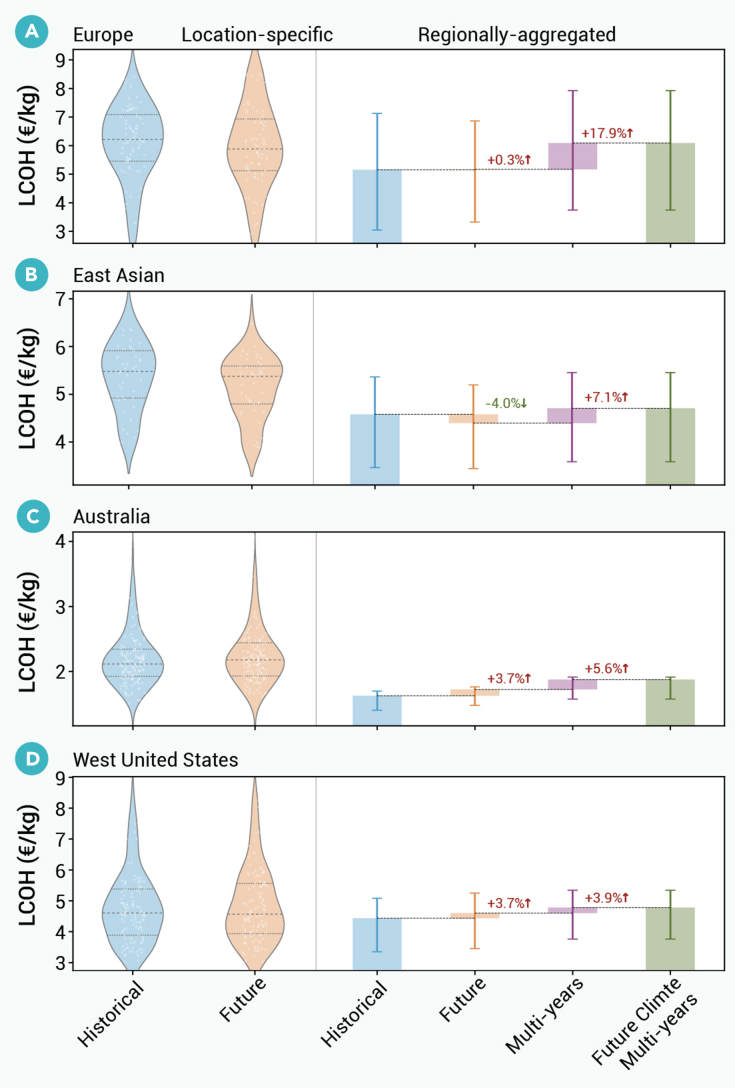
Regional comparison of climate change and internal variability effects on location-specific and regionally aggregated hydrogen production costs Location-specific (left) and regionally aggregated (right) H_2_ production LCOHs for four regions: (A) Europe, (B) East Asia, (C) Australia, and (D) Western United States. For each region, the violin plots (left) show location-specific LCOHs under historical and future climates, where dot distribution means different grid cells in the region. The waterfall chart (right) illustrates the regionally aggregated LCOH under the historical climate, change to future climate, and change to future climate while simultaneously optimization for 10-year period (2065–2075). Error bars indicate variability across sub-regions within each region (e.g., across states in the Western United States). Dashed lines in the violin plots indicate the 25th, 50th, and 75th percentiles.

We now focus on four regions that will likely feature large-scale hydrogen production in the future: Europe, Australia, East Asia, and the western United States. Figure 7 compares LCOHs under historical and future climate conditions, then compares the effect of climate change on hydrogen costs relative to the effect of considering historical internal variability (i.e., of considering multiple historical weather years). The method for capturing internal variability is described in section hydrogen-system investment and operation model. In all regions, accounting for internal variability has a larger effect on LCOHs than climate change. In Europe, for instance, climate change increases the LCOH by 0.3% on average across locations, while accounting for internal variability increases the LCOH by 18% on average. Internal variability increases LCOH by increasing investment needs to reflect differences in weather years (illustrated in Figure S8), typically through changes in investments in hydrogen storage to account for inter-annual variability (shown in Figure S10). In East Asia, climate change decreases the LCOH by roughly 5% on average across sites, but, if accounting for internal variability under future climate, the LCOH increases by 7%. Thus, the effect of climate change is roughly on par with the magnitude of historical internal variability.

## Discussion

Hydrogen will play a key role in decarbonizing economic sectors and economies around the world, with most hydrogen deployment expected to occur between 2035 and 2050. We use a dynamic system-optimization model to analyze hydrogen costs and system configurations for all global locations under future climate change. We find that climate change will generally have a small effect on hydrogen costs and system configurations, particularly in locations where LCOHs are small and where, therefore, hydrogen deployment is more likely to occur. At locations with low LCOHs, climate change tends to change LCOHs by less than 10% (Figure 3). At locations with higher LCOHs, the impact of climate change can be as large as 30%. Optimal hydrogen-system configurations, whether using grid-connected or local renewables, largely do not change when planning for a future climate, with the exception of generally increasing storage needs on the order of 20% (Figure 4). If we consider regionally integrated (i.e., grid-connected) rather than location-specific hydrogen production, the effect of climate change is even more modest.

Overall, our results indicate that climate change will have a modest effect on future hydrogen costs and system configurations. Thus, analyses that have found hydrogen could play a key role in global decarbonization using historical weather data would reach largely similar conclusions using future weather data. However, these changes could still be important under specific conditions, such as international hydrogen trading, where hydrogen cost differences are a key driving factor. For instance, climate change reduces cost differences between Australia and East Asia, an expected export-import pair,^29^ by up to 14%.

We compare the effect of climate change to the effect of accounting for internal variability in the current climate and find the latter has a more significant effect on LCOHs than the former (Figure 7). These results underscore the need to capture the influence of diverse weather years on hydrogen design and costs and suggest that using large amounts of historical weather data is more important than capturing future weather. Much of the prior research suggests climate change will have modest effects on wind and solar resources, but inter-annual variability in wind and solar can be significant.^30^^,^^31^ Our results reflect these trends in the literature. Also, for near-term green-hydrogen project finance, managing the significant inter-annual variability is important for de-risking investments. For long-term hydrogen infrastructure planning, the persistent, directional signal of climate change is also important for strategic decision-making.

We compare LCOHs and hydrogen-system configurations between our dynamic optimization model and simple LCOE-based models, which are common among global hydrogen analyses.^8^^,^^14^ We found the simple LCOE-based method can significantly underestimate LCOH costs, including at sites with moderate LCOH costs, by up to 40%. Using dynamic optimization models is therefore crucial to accurately understand the global potential for and cost distribution of hydrogen deployment, which in turn can inform the role of hydrogen in decarbonization. Our system-optimization model can efficiently process large volumes of locations and weather data. The computation time for all global locations with 1° × 1° spatial resolution, and one weather year is roughly 6 h, providing a scalable and tractable method for future global hydrogen analyses.

Our analysis has several limitations. First, for the future climate scenarios, we only focus on an extreme climate realization (SSP585) to capture the upper bound of potential climate change impacts. Even at this extreme climate realization, our results demonstrate modest effects of climate change, suggesting even more modest effects under milder climate scenarios. Second, our analysis focuses on how changes in wind and solar resources drive changes in LCOH costs. Climate change can affect hydrogen production through other means, e.g., through increasing water scarcity, a necessary input for electrolysis. Third, climate change can impact the full hydrogen value chain, including transportation (e.g., via extreme weather events affecting shipping and infrastructure) and storage facilities. A full analysis of these impacts would require different modeling approaches and is a valuable avenue for future research, while our study provides a necessary and foundational analysis of the production stage, which is most directly and continuously affected by the shifting wind and solar resource patterns that are a primary consequence of climate change. Finally, this study focuses on a rigorous analysis of how climate change will affect green hydrogen, while future research could provide a more comprehensive analysis by integrating the impacts of both climate change and environmental externalities on the cost-competitiveness of green hydrogen. Despite these limitations, our study suggests that the lack of prior analyses on hydrogen costs and system design under future climate change should not change our understanding of hydrogen as a key decarbonization technology. Climate change will have a small effect on LCOHs and ultimately prove less important to future hydrogen costs than other factors, such as internal climate variability.

## Resource availability

### Materials availability

This study did not generate new unique materials/reagents.

### Data and code availability

• The input climate data for eight CMIP6 GCMs are derived from Lawrence Livermore National Laboratory.
• The calculated wind and solar capacity factor series from eight GCMs, from historical to future climate scenarios, are available from the corresponding author on request.
• Other data are included in the manuscript and/or supplemental information.
• This study did not generate new software.

## Funding and acknowledgments

We thank the 10.13039/501100001809National Natural Science Foundation of China under grants 72595830/72595831 and 72571007. We thank the US National Science Foundation under award number 2142421 for funding (to M.T.C.). We thank Advanced Research Computing at the University of Michigan, Ann Arbor, for high-performance computing and storage resources. The funders had no role in study design, data collection and analysis, decision to publish, or preparation of the manuscript.

## Author contributions

H.W., M.S., and M.T.C. designed the research. H.W., M.S., and M.T.C. performed the research. H.W., M.S., and M.T.C. wrote the original draft. M.T.C. and M.S. acquired the financial support for the project. M.T.C. and M.S. reviewed and edited the final manuscript. All authors contributed to the discussions on the framework and the editing of this article.

## Declaration of interests

The authors declare no competing interests.

## Declaration of generative AI and AI-assisted technologies in the writing process

During the editing of the original draft, the authors used GitHub Copilot in Visual Studio Code for language readability improvement. After using this tool, the authors have carefully checked the manuscript and are fully responsible for the content of the publication.

## Supplemental information

Document S1. Figures S1–S14, Tables S1 and S2, and Notes S1–S4

Document S2. Article plus supplemental information

## References

[1] (2024). World Energy Outlook 2024 – Analysis. https://www.iea.org/reports/world-energy-outlook-2024.

[2] (2023). World Energy Transitions Outlook 2023: 1.5°C Pathway. https://www.irena.org/Publications/2023/Jun/World-Energy-Transitions-Outlook-2023.

[3] Odenweller A., Ueckerdt F., Nemet G.F. (2022). Probabilistic feasibility space of scaling up green hydrogen supply. Nat. Energy.

[4] (2024). Global Hydrogen Review 2024 – Analysis. https://www.iea.org/reports/global-hydrogen-review-2024.

[5] Ueckerdt F., Bauer C., Dirnaichner A. (2021). Potential and risks of hydrogen-based e-fuels in climate change mitigation. Nat. Clim. Change.

[6] McKinsey Global Energy Perspective 2023: Hydrogen outlook — McKinsey. https://www.mckinsey.com/industries/oil-and-gas/our-insights/global-energy-perspective-2023-hydrogen-outlook.

[7] Ueckerdt F., Verpoort P.C., Anantharaman R. (2024). On the cost competitiveness of blue and green hydrogen. Joule.

[8] de Kleijne K., Huijbregts M.A.J., Knobloch F. (2024). Worldwide greenhouse gas emissions of green hydrogen production and transport. Nat. Energy.

[9] Brandt J., Iversen T., Eckert C. (2024). Cost and competitiveness of green hydrogen and the effects of the European Union regulatory framework. Nat. Energy.

[10] Song S., Lin H., Sherman P. (2021). Production of hydrogen from offshore wind in China and cost-competitive supply to Japan. Nat. Commun..

[11] Tonelli D., Rosa L., Gabrielli P. (2023). Global land and water limits to electrolytic hydrogen production using wind and solar resources. Nat. Commun..

[12] Mingolla S., Gabrielli P., Manzotti A. (2024). Effects of emissions caps on the costs and feasibility of low-carbon hydrogen in the European ammonia industry. Nat. Commun..

[13] Terlouw T., Rosa L., Bauer C. (2024). Future hydrogen economies imply environmental trade-offs and a supply-demand mismatch. Nat. Commun..

[14] Tonelli D., Rosa L., Gabrielli P. (2024). Cost-competitive decentralized ammonia fertilizer production can increase food security. Nat. Food.

[15] Pan G., Gu W., Hu Q. (2021). Cost and low-carbon competitiveness of electrolytic hydrogen in China. Energy Environ. Sci..

[16] Bracci J.M., Sherwin E.D., Boness N.L. (2023). A cost comparison of various hourly-reliable and net-zero hydrogen production pathways in the United States. Nat. Commun..

[17] Terlouw T., Bauer C., McKenna R. (2022). Large-scale hydrogen production via water electrolysis: A techno-economic and environmental assessment. Energy Environ. Sci..

[18] Yalew S.G., van Vliet M.T.H., Gernaat D.E.H.J. (2020). Impacts of climate change on energy systems in global and regional scenarios. Nat. Energy.

[19] Lei Y., Wang Z., Wang D. (2023). Co-benefits of carbon neutrality in enhancing and stabilizing solar and wind energy. Nat. Clim. Change.

[20] Liu L., He G., Wu M. (2023). Climate change impacts on planned supply–demand match in global wind and solar energy systems. Nat. Energy.

[21] Kapica J., Jurasz J., Canales F.A. (2024). The potential impact of climate change on european renewable energy droughts. Renew. Sustain. Energy Rev..

[22] Yin J., Molini A., Porporato A. (2020). Impacts of solar intermittency on future photovoltaic reliability. Nat. Commun..

[23] Wu H., Qiu D., Zhang L. (2024). Adaptive multi-agent reinforcement learning for flexible resource management in a virtual power plant with dynamic participating multi-energy buildings. Appl. Energy.

[24] Wu H., Chen J., Vaishnav P. (2026). Technological improvements in EV batteries offset climate-induced durability challenges. Research Square.

[25] Wu H., Kong Q., Huber M. (2026). Climate change will increase high temperature risks, degradation, and costs of rooftop photovoltaics globally. Joule.

[26] Wang C., Song J., Shi D. (2023). Impacts of climate change, population growth, and power sector decarbonization on urban building energy use. Nat. Commun..

[27] Vuichard N., Papale D. (2015). Filling the gaps in meteorological continuous data measured at FLUXNET sites with ERA-Interim reanalysis. Earth Syst. Sci. Data.

[28] Müller L.A., Leonard A., Trotter P.A. (2023). Green hydrogen production and use in low-and middle-income countries: A least-cost geospatial modelling approach applied to kenya. Appl. Energy.

[29] IRENA - Global hydrogen trade. https://www.irena.org/Energy-Transition/Technology/Hydrogen/Global-hydrogen-trade.

[30] Perera A.T.D., Nik V.M., Chen D. (2020). Quantifying the impacts of climate change and extreme climate events on energy systems. Nat. Energy.

[31] Ruggles T.H., Virgüez E., Reich N. (2024). Planning reliable wind-and solar-based electricity systems. Adv. Appl. Energy.

