# Supplementary material for: Updating global green-hydrogen production costs and configurations under future climates
Source: Innovation (Camb). 2026 Feb 5;7(3):101303. doi: 10.1016/j.xinn.2026.101303 (PMC12957562; doi:10.1016/j.xinn.2026.101303)
Supplement: Document S1. Figures S1–S14, Tables S1 and S2, and Notes S1–S4 [file mmc1.pdf]

The Innovation, Volume 7

Supplemental Information

Updating global green-hydrogen production costs and configurations under future climates

Haochi Wu, Mingyang Sun, and Michael T. Craig

The Innovation, Volume 7

Supplemental Information

Updating global green-hydrogen production costs and configurations under future climates

Haochi Wu, Mingyang Sun, and Michael T. Craig

Supplementary Information

S1.1 Supplementary Note 1: H2 investment model

The parameters are listed in Table S2[S1,S2]. Also, please note that we are using a fixed cost parameter in contemporary to isolate other impacts and focus on the impact from climate changes and LCOH. But it is feasible to roughly approximate the LCOH in mid-century with future cost parameter using scalar factor, as reduction pathway shown in Figure S11. An overlapped reduction range could be about 0.6, and given that the system cost is mainly made up of the capital and operational cost of these main components of the hybrid green hydrogen system, including solar PV, wind Turbine, electrolyzer, and storage (Figure S10). It is important to note that a uniform cost reduction across all technologies (e.g., a factor of 0.6) would scale the absolute LCOH but leave the relative change due to climate (ΔLCOH%) unchanged. To test the robustness of this finding against uneven cost reductions, we performed a sensitivity analysis (see Figure S17). While the absolute LCOH would decrease significantly with lower technology costs, the relative change in LCOH due to climate change (the ΔLCOH) is stable. Across all technology cost scenarios and regions, the climate-induced ΔLCOH deviates by less than 0.5 percentage points from our baseline findings. This analysis provides strong evidence that even as hydrogen technologies become cheaper, the regional shifts in competitiveness and investment risk driven by climate change will persist.

$$\sum_t M_E(t) \cdot \leq \sum_t D_{H_2}(t) \cdot \epsilon_{H_2} \quad \text{(External firm electricity constraint)} \tag{1}$$

Where $M_E(t)$ is the imported grid electricity at time t (MWh), $D_{H_2}(t)$ is the hydrogen demand at time t (kg H_2/h), and ϵ_{H_2} is the external firm electricity limit per kilogram of hydrogen produced (MWh/kg H_2).

$$V_{E,\mathrm{PV}}(t) = s(t) \cdot \eta_{\mathrm{PV}} \cdot P_{\mathrm{PV}} \quad \forall t \quad \text{(Solar PV generation)} \tag{2}$$

Where $V_{E,\mathrm{PV}}(t)$ is the electricity generated from solar PV at time t (MWh), $s(t)$ is the solar capacity factor at time t, η_{PV} is the efficiency of the solar PV system, and P_{PV} is the installed capacity of solar PV (MW).

$$V_{E,\mathrm{WT}}(t) = w(t) \cdot \eta_{\mathrm{WT}} \cdot P_{\mathrm{WT}} \quad \forall t \quad \text{(Wind turbine generation)} \tag{3}$$

Where $V_{E,\mathrm{WT}}(t)$ is the electricity generated from wind turbines at time t (MWh), $w(t)$ is the wind capacity factor at time t, η_{WT} is the efficiency of the wind turbines, and P_{WT} is the installed capacity of wind turbines (MW).

$$S_{E,B}(t) = (1-\lambda_B)S_{E,B}(t-1) + \eta_{c,B}U_{E,B}(t) - \frac{V_{E,B}(t)}{\eta_{d,B}} \quad \forall t \quad \text{(Battery storage balance)} \tag{4}$$

Where $S_{E,B}(t)$ is the energy stored in batteries at time t (MWh), λ_B is the self-discharge rate of the batteries, $\eta_{c,B}$ is the charging efficiency, $U_{E,B}(t)$ is the electricity input to the batteries at time t (MWh), $V_{E,B}(t)$ is the electricity output from the batteries at time t (MWh), and $\eta_{d,B}$ is the discharging efficiency.

$$S_{E,B}(t) \leq P_B \quad \forall t \quad \text{(Battery capacity limit)} \tag{5}$$

Where P_B is the installed battery capacity (MWh).

$$U_{E,B}(t) \leq \frac{P_B}{\tau_{c,B}} \quad \forall t \quad \text{(Battery charge rate limit)} \tag{6}$$

Where $\tau_{c,B}$ is the charging time constant of the batteries (h).

$$V_{E,B}(t) \leq \frac{P_B}{\tau_{d,B}} \quad \forall t \quad \text{(Battery discharge rate limit)} \tag{7}$$

Where $\tau_{d,B}$ is the discharging time constant of the batteries (h).

$$U_{E,\text{EL}}(t) \leq P_{\text{EL}} \quad \forall t \quad \text{(Electrolyzer capacity limit)} \tag{8}$$

Where $U_{E,\text{EL}}(t)$ is the electricity input to the electrolyzers at time t (MWh) and P_{EL} is the installed capacity of electrolyzers (MW).

$$V_{H_2,\text{EL}}(t) = \eta_{\text{EL}} \cdot U_{E,\text{EL}}(t) \quad \forall t \quad \text{(Hydrogen production)} \tag{9}$$

Where $V_{H_2,\text{EL}}(t)$ is the hydrogen produced by the electrolyzers at time t (kg H_2/h) and η_{EL} is the efficiency of the electrolyzers.

$$U_{E,\text{CP}}(t) \cdot \eta_{\text{CP}} \leq P_{\text{CP}} \quad \forall t \quad \text{(Compressor capacity limit)} \tag{10}$$

Where $U_{E,\text{CP}}(t)$ is the electricity input to the compressors at time t (MWh), η_{CP} is the efficiency of the compressors, and P_{CP} is the installed capacity of the compressors (kg H_2/h).

$$V_{H_2,\text{CP}}(t) = \eta_{\text{CP}} \cdot U_{E,\text{CP}}(t) \quad \forall t \quad \text{(Compressed hydrogen output)} \tag{11}$$

Where $V_{H_2,\text{CP}}(t)$ is the compressed hydrogen output at time t (kg H_2/h).

$$V_{H_2,\text{EL}}(t) \geq U_{H_2,\text{CP}}(t) \quad \forall t \quad \text{(Hydrogen input to compressor)} \tag{12}$$

Where $U_{H_2,\text{CP}}(t)$ is the hydrogen input to the compressors at time t (kg H_2/h).

$$S_{H_2,\text{ST}}(t) = S_{H_2,\text{ST}}(t-1) + U_{H_2,\text{ST}}(t) - V_{H_2,\text{ST}}(t) \quad \forall t \quad \text{(Hydrogen storage balance)} \tag{13}$$

Where $S_{H_2,\text{ST}}(t)$ is the hydrogen stored in tanks at time t (kg H_2), $U_{H_2,\text{ST}}(t)$ is the hydrogen input to storage tanks at time t (kg H_2/h), and $V_{H_2,\text{ST}}(t)$ is the hydrogen output from storage tanks at time t (kg H_2/h).

$$S_{H_2,\text{ST}}(t) \leq P_{\text{ST}} \quad \forall t \quad \text{(Storage tank capacity)} \tag{14}$$

Where P_{ST} is the capacity of hydrogen storage tanks (kg H_2).

$$M_E(t) + V_{E,\text{PV}}(t) + V_{E,\text{WT}}(t) - U_{E,B}(t) + V_{E,B}(t) \geq U_{E,\text{EL}}(t) + U_{E,\text{CP}}(t) \quad \forall t \quad \text{(Electricity balance)} \tag{15}$$

Where $M_E(t)$ is the imported grid electricity at time t (MWh), $V_{E,\text{PV}}(t)$ is the solar PV generation (MWh), $V_{E,\text{WT}}(t)$ is the wind turbine generation (MWh), $U_{E,B}(t)$ is the electricity input to batteries (MWh), $V_{E,B}(t)$ is the electricity output from batteries (MWh), $U_{E,\text{EL}}(t)$ is the electricity input to electrolyzers (MWh), and $U_{E,\text{CP}}(t)$ is the electricity input to compressors (MWh).

$$V_{H_2,\mathrm{EL}}(t) - U_{H_2,\mathrm{ST}}(t) + V_{H_2,\mathrm{ST}}(t) = D_{H_2}(t) \quad \forall t \quad \text{(Hydrogen demand fulfillment)} \tag{16}$$

Where $V_{H_2,\mathrm{EL}}(t)$ is the hydrogen produced (kg H_2/h), $U_{H_2,\mathrm{ST}}(t)$ is the hydrogen input to storage (kg H_2/h), $V_{H_2,\mathrm{ST}}(t)$ is the hydrogen output from storage (kg H_2/h), and $D_{H_2}(t)$ is the hydrogen demand at time t (kg H_2/h).

$$D_{H_2}^{\min} \leq D_{H_2}(t) \leq D_{H_2}^{\max} \quad \forall t \quad \text{(Hydrogen production limits)} \tag{17}$$

Where $D_{H_2}^{\min}$ and $D_{H_2}^{\max}$ are the minimum and maximum hydrogen production levels (kg H_2/h), depending on production type.

$$\sum_t D_{H_2}(t) \geq \text{Annual } \mathrm{H_2} \text{ demand} \quad \text{(Total hydrogen demand)} \tag{18}$$

Table S1: Literature Review on Hydrogen Production

Year	Source	Research Question	Hybrid Plant Dynamics	Geographic Mapping	Climate Change
2021	[S3]	Solar Green Hydrogen Benefits by Provinces in China	✓		
2021	[S4]	China's offshore hydrogen production		✓	
2023	[S5]	The cost of reliable hydrogen generation at the hourly level	✓		
2023	[S6]	Land and water limitations, renewable hydrogen production		✓	
2022	[S7]	The trade-off between H2 cost and carbon emissions in a mixed hydrogen system	✓		
2024	[S8]	Green hydrogen LCA carbon footprint		✓	
2024	[S9]	Trade-off cost and carbon of H2 in Europe	✓		
2024	[S1]	Cost competitiveness of hydrogen demand for continuous nitrogen fertilizer		✓	
2024	[S10]	Cost and environmental trade-off of green hydrogen with demand-supply match fertilizer		✓	
2024	[S2]	Cost of hydrogen based Ammonia generation	roughly sampled	✓	
N/A	Our study	Cost and footprint changes in hydrogen production under climate change	✓	✓	✓

S1.2 Supplementary Note 2: Capacity factor series for wind and solar

The wind capacity factor is calculated as follows[S11]:

$$W_{100} = W_{10} \left(\frac{100\,\mathrm{m}}{10\,\mathrm{m}} \right)^{1/7} \tag{19}$$

$$\rho_d = \frac{p}{RT} \tag{20}$$

where $R = 287.058\,\mathrm{J\,kg^{-1}\,K^{-1}}$.

Table S2: Input Parameters for H2 system model[S1]

Parameter	Description	Unit	Value
c_{EL}	cost for electrolyzer	EUR	1637576.0
OM_{EL}	Operation and Maintenance cost for electrolyzer	-	0.520
c_B	Unit cost utility-scale Li-ion battery system	EUR/MWh	608082.0
p_{ETS}	Price of ETS	EUR/ton CO_2	2.23561
c_{PV}	cost for utility-scale photovoltaics	EUR/MW	580749.0
c_{WT}	cost for utility-scale wind turbines	EUR/MW	993424.7938
p_E	Price of grid electricity	EUR/MWh	5317.8761532
$\text{lifetime}_{H2\_discount}$	Lifetime production discounted	-	1708200000.0
OM_B	Operation and Maintenance cost for batteries	-	0.650
OM_{PV}	Operation and Maintenance cost for photovoltaics	-	449098.0
OM_{WT}	Operation and Maintenance cost for wind turbines	-	661830.0
c_{CP}	Unit cost of compression system	EUR	26591.0
OM_{CP}	Operation and Maintenance cost for compression system	-	1.040
c_{ST}	Unit cost of H_2 storage tanks	EUR/kg H_2 stored	455.0
T_{LVAC}	Low-voltage alternating current transmission cost	EUR	14000000.0
T_{HVAC}	High-voltage alternating current transmission cost	EUR	33000000.0
c_{HVAC_wires}	Cost of HVAC wires	EUR	1600000.0
$COST_{RETROFIT}$	Retrofit cost	EUR	4000000.0

$$\rho_m = \rho_d \frac{1+q}{1+1.609q} \tag{21}$$

$$W_{100} = W_{100}\left(\frac{\rho_m}{1.225}\right)^{1/3} \tag{22}$$

$$P = f(W_{100}) \tag{23}$$

$$P = \begin{cases} 0\,\text{kW}, & W_{100} < 2.5\,\text{m}\,\text{s}^{-1} \\ -0.05\,W_{100}^5 + 1.24\,W_{100}^4 - 9.74\,W_{100}^3 + 45.32\,W_{100}^2 - 78.08\,W_{100} + 35.62, & 2.5\,\text{m}\,\text{s}^{-1} \leq W_{100} \leq 12\,\text{m}\,\text{s}^{-1} \\ 1,500\,\text{kW}, & 12\,\text{m}\,\text{s}^{-1} < W_{100} \leq 25.5\,\text{m}\,\text{s}^{-1} \\ 0\,\text{kW}, & W_{100} > 25.5\,\text{m}\,\text{s}^{-1} \end{cases} \tag{24}$$

$$\text{CF} = \frac{P}{P_{\text{rated}}} \tag{25}$$

where $P_{\text{rated}} = 1,500$ kW is the rated power of the turbine.

The wind profile is shown in Figure S13.

The solar capacity factor is calculated as follows[S12]:

$$PV_{\text{pot}}(t) = P_R(t)\frac{\text{RSDS}(t)}{\text{RSDS}_{\text{STC}}} \tag{26}$$

where RSDS_{STC} denotes to solar irradiance under standard test conditions (equals to 1,000 W/m^2), and performance ratio P_R refers to PV cell efficiency under different cell temperatures and is defined in Equation (27).

$$P_R(t) = 1 + \gamma \left[T_{\text{cell}}(t) - T_{\text{STC}} \right] \tag{27}$$

where T_{cell} refers to PV cell temperature, T_{STC} denotes to cell temperarture under standard test conditions (equals to 25°C), and γ is the temperature coefficient (equals to $-0.005°\text{C}^{-1}$), representing c-Si PV panels.

$$T_{\text{cell}}(t) = c_1 + c_2\text{TAS}(t) + c_3\text{RSDS}(t) + c_4\text{VWS}(t) \tag{28}$$

with $c_1 = 4.3°\text{C}$, $c_2 = 0.943$, $c_3 = 0.028\,\text{C m}^2\text{W}^{-1}$, and $c_4 = -1.528\,\text{C sm}^{-1}$.

S1.3 Supplementary Note 3: Spatial aggregation

The administrative boundary shapefiles for each country are obtained from 'Natural Earth' public map dataset and processed using the Python package 'geopanda'. For six specific countries, namely the United States, Canada, China, Australia, Russia, and Brazil, we refined the boundaries to secondary administrative units due to their larger geographical spans.

Specifically, the process of aggregating renewable energy data involves several steps. First, we create a global administrative boundary shape using the 'create global adm shape' function. Next, we load the climate data and adjust the longitude to ensure it is in increasing order. We then rename the dimensions and coordinates for consistency and calculate the mean over time.

To obtain the matrix for different shapes, we use the 'create shape matrix' function, which generates a matrix based on the solar capacity factor (CF) and the administrative boundaries. This matrix is then plotted for visualization.

We select the top 20% of grid cells based on the mean solar CF using the 'select top grid' function. This selection represents areas with high resource potential. However, the top 20% does not necessarily represent 20% of the actual land area due to potential issues with land availability and feasibility. This selection is also plotted for visualization. The selected matrix is saved for future use.

For each administrative shape, we calculate the mean CF by aggregating the matrix using the aggregate matrix function. If required, we normalize the results per unit capacity. The mean CF values are then attributed to the administrative boundaries and plotted.

Finally, we create a time series for each administrative shape by reloading the climate data and repeating the aggregation process. The results are saved in a NetCDF file for further analysis. The mathematical equation for the above analysis could be summarized as follows:

$$\begin{aligned} \text{Solar\_agg}(t) &= \frac{1}{i} \cdot \sum_{i \in \text{TopSolar}} \text{Solar}_i(t) \\ \text{Wind\_agg}(t) &= \frac{1}{j} \cdot \sum_{j \in \text{TopWind}} \text{Wind}_j(t) \end{aligned} \tag{29}$$

Where $\text{Solar}_i(t)$ and $\text{Wind}_j(t)$ represent the solar and wind output at location i and j at time t, respectively. i and j are the locations numbers selected in the specific region.

S1.4 Supplementary Note 4: Comparative analysis for optimization-based method and simplified method

To systematically investigate the drivers of the discrepancy between the simplified LCOE-based model and our dynamic optimization model, we performed a multivariate regression analysis. The analysis employed an Ordinary Least Squares (OLS) model, implemented using the statsmodels library in Python, with the absolute LCOH error (€/kg) as the dependent variable. We selected six independent predictor variables categorized into two groups: four Optimized System Design Parameters (Renewable Oversizing Ratio, H2 Storage Duration, Solar Ratio, and Electrolyzer Utilization Ratio) and two Climate Resource Endowments (Annual Solar CF and Annual Wind CF).

To ensure the direct comparability of the coefficients and thus ascertain the relative importance of each predictor, all six independent variables were standardized to a mean of zero and a standard deviation of one prior to model fitting. The resulting model explains 88.2% of the variance in the LCOH error (R^2 = 0.882) and yields several key insights, as shown by the standardized coefficients (Figure S15).

First, a higher renewable oversizing ratio and longer H2 storage duration are both strong predictors of a negative error, indicating that the simplified model systematically underestimates costs in systems that our optimization model designs with large capital investments to ensure reliability. Conversely, a higher electrolyzer utilization ratio and a larger share of solar PV in the portfolio are associated with a positive error, signifying that the simplified model overestimates the LCOH for highly efficient systems where the optimization model can effectively balance less variable resources. Finally, better underlying renewable potential, reflected by higher annual capacity factors for both wind and solar, is a strong predictor of a negative error. This effect is particularly pronounced for wind, suggesting that while the simplified model naively translates high resource availability into low costs, our optimization model correctly identifies the need for substantial system-level investment to reliably harness that potential.

To visualize the unique effect of each standardized predictor, we use partial regression plots (Figure S16), which isolate each predictor's relationship with the LCOH error after statistically controlling for the influence of all other variables, providing a clear graphical confirmation of each factor's independent impact.

S1.5 Supplementary Figures

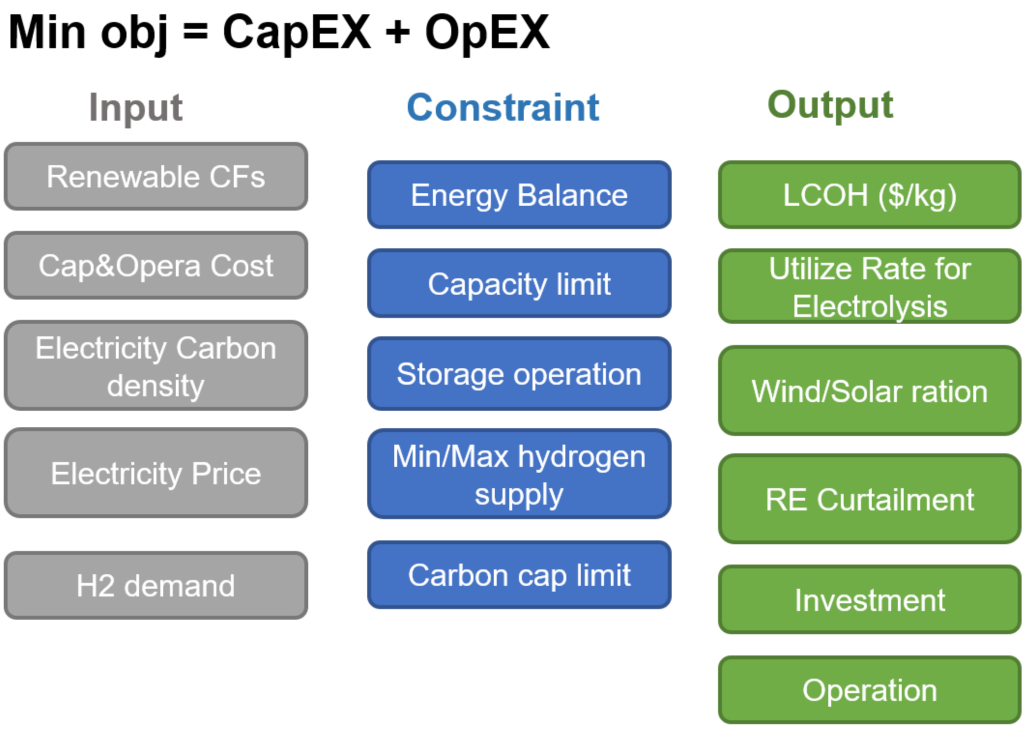

Figure S1: Schematic for the modeled hydrogen system. The system consists of renewable energy sources, an electrolyzer, a storage system. And the system is optimized based on the mathematical formulation

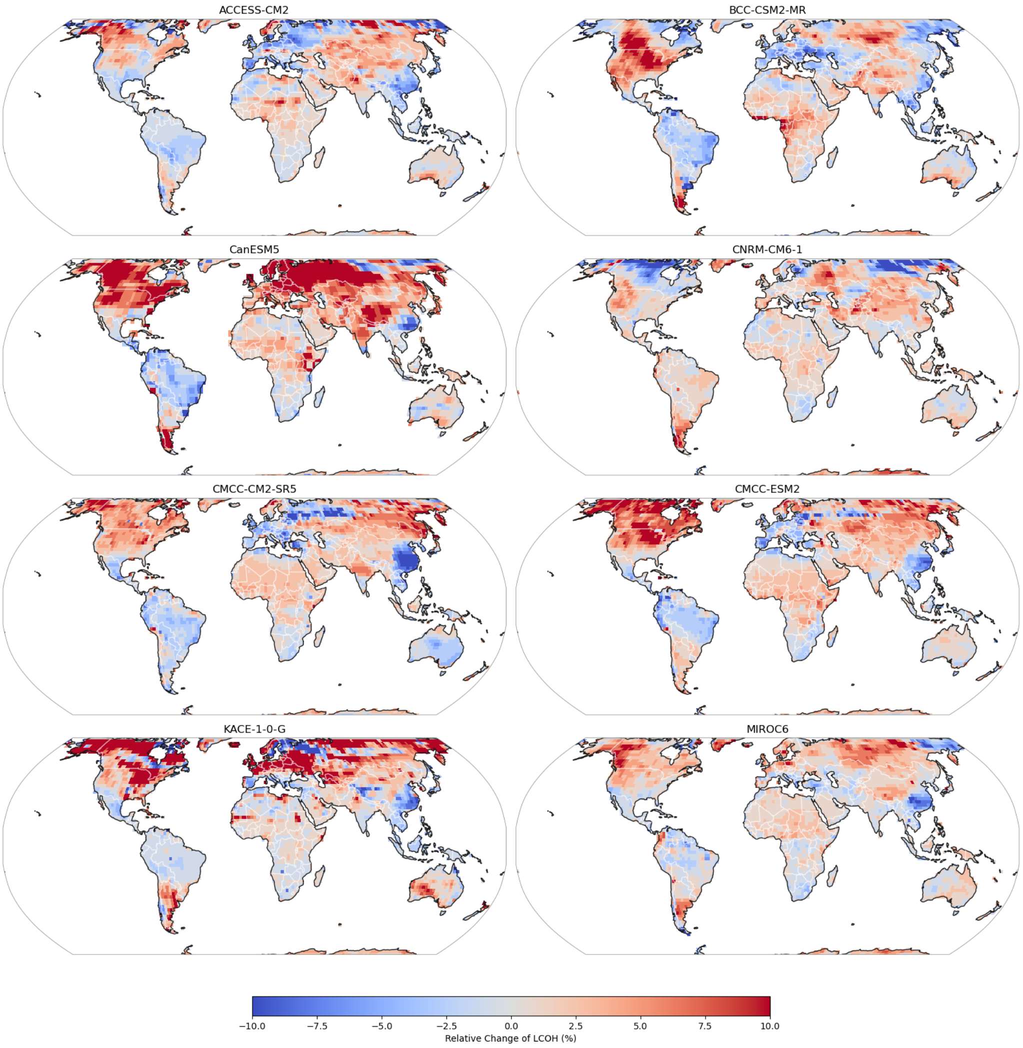

Figure S2: Change in LCOH under 8 GCMs for global locations. The comparison is made between the historical period (2000-2010) and the future period (2065-2075). The climate sensitivity for GCMs is observed to be positively related to the change in LCOH. For example, models with higher ECS tend to have relatively higher LCOH change, like CanESM5, and vice versa for MIROC6.

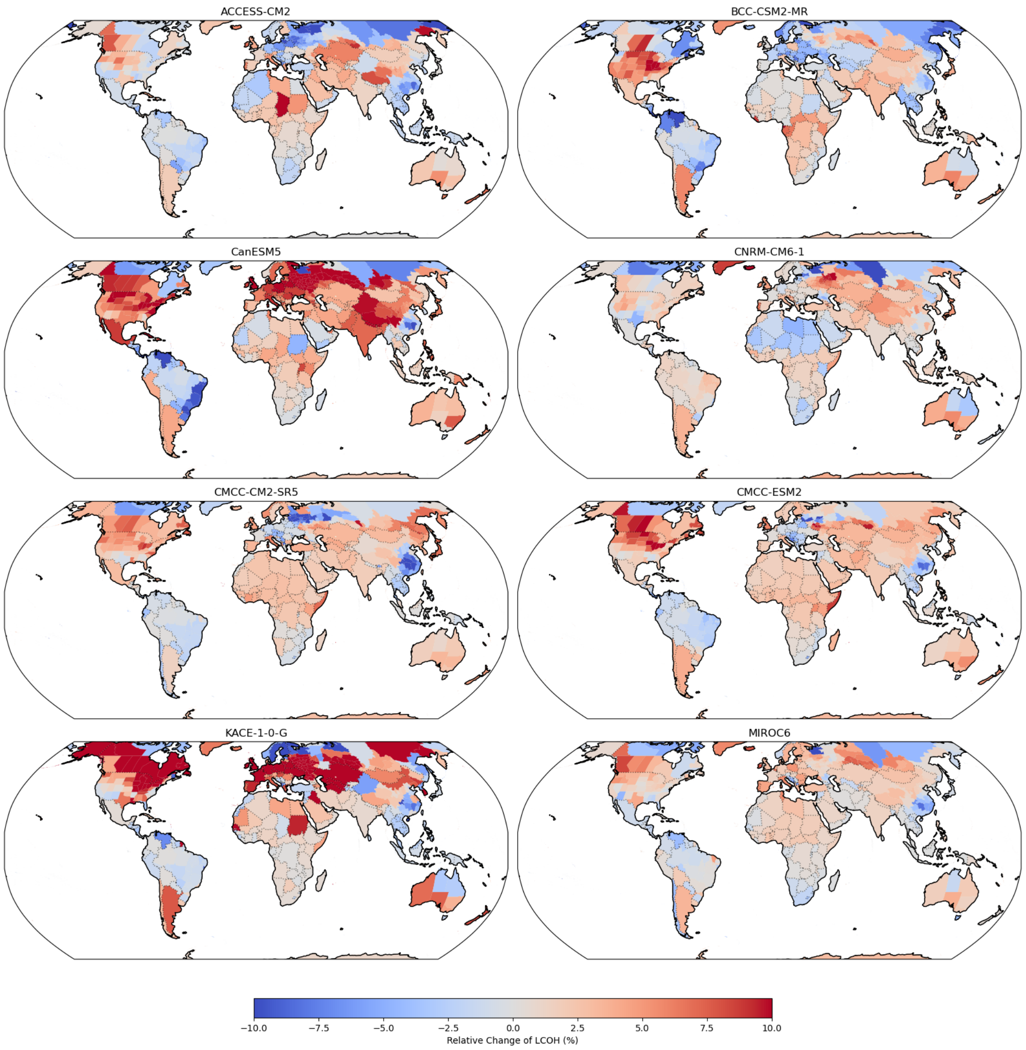

Figure S3: Change in LCOH under 8 GCMs for global regions. The comparison is made between the historical period (2000-2010) and the future period (2065-2075). Similarly to locational result, the climate sensitivity for GCMs is observed to be positively related to the change in LCOH.

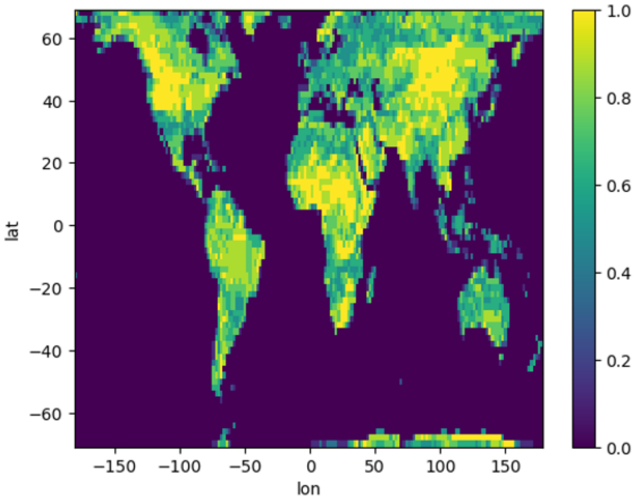

Figure S4: Color shows the agreement fraction in each location. The threshold for model agreement is 0.75, meaning 6 out of 8 models agreed on the average change direction.

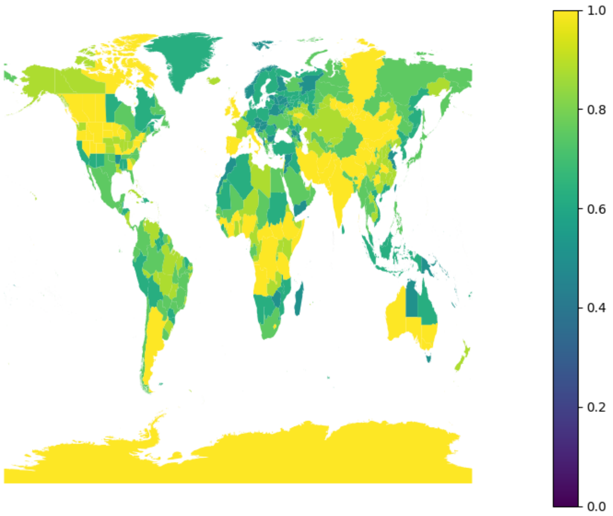

Figure S5: Color shows agreement fraction in each region. The threshold for model agreement is 0.75, meaning 6 out of 8 models agreed on the average change direction.

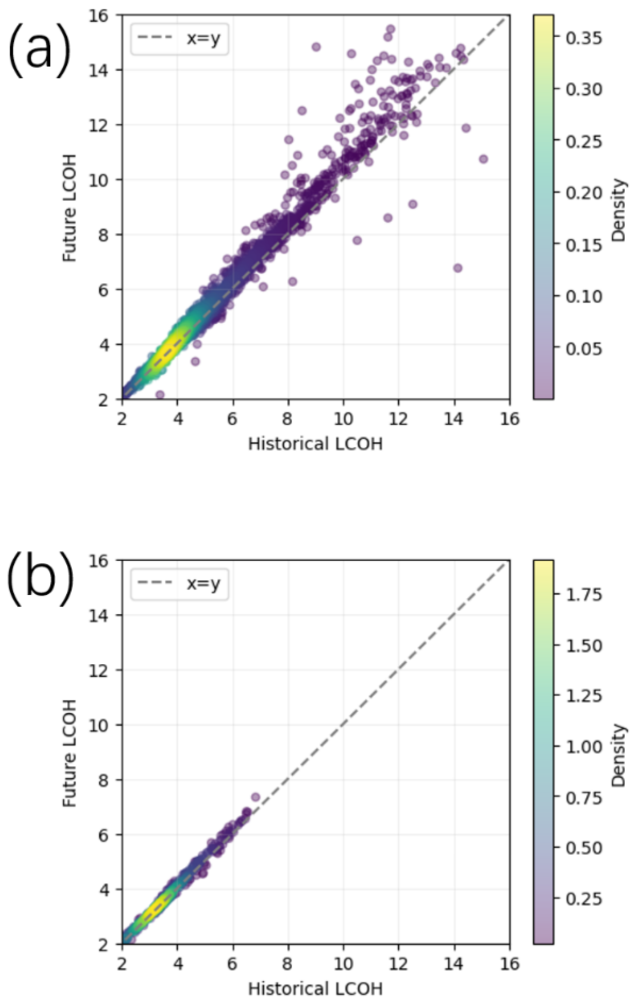

Figure S6
Scatter plot of LCOH for the historical period (2000-2010) and the future period (2065-2075) with KDE density estimation. (a) Locational based renewable energy, and (b) Regional aggregated renewable energy.

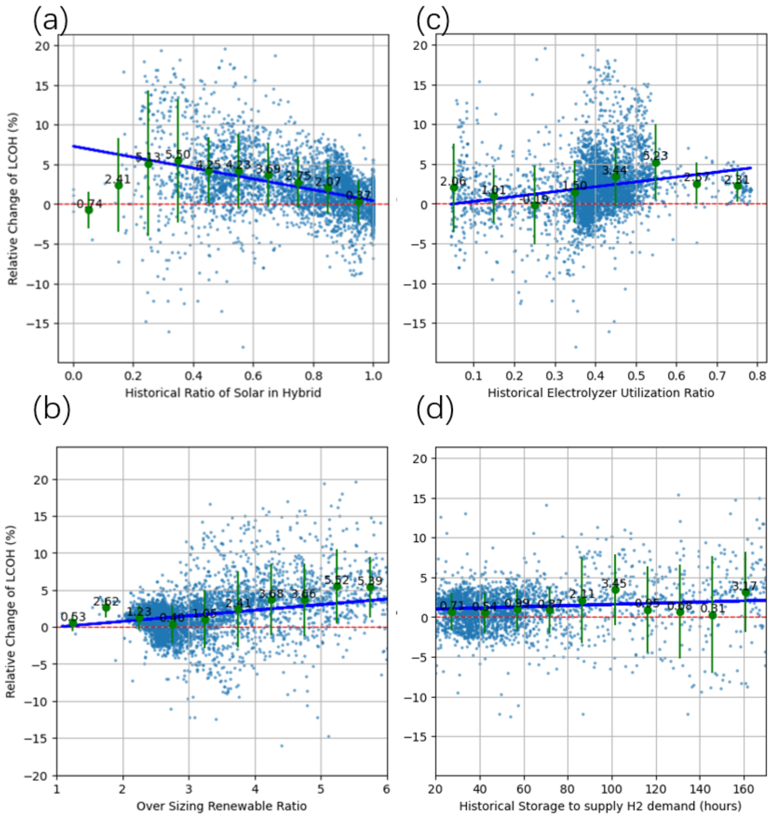

Figure S7: The relationship between Δ LCOH and system design parameters: (a) ratio of solar in renewable energy, (b) Over sizing ratio of renewable to electrolyser capacity, (c) electrolyser Utilization Ratio and (d) storage duration.

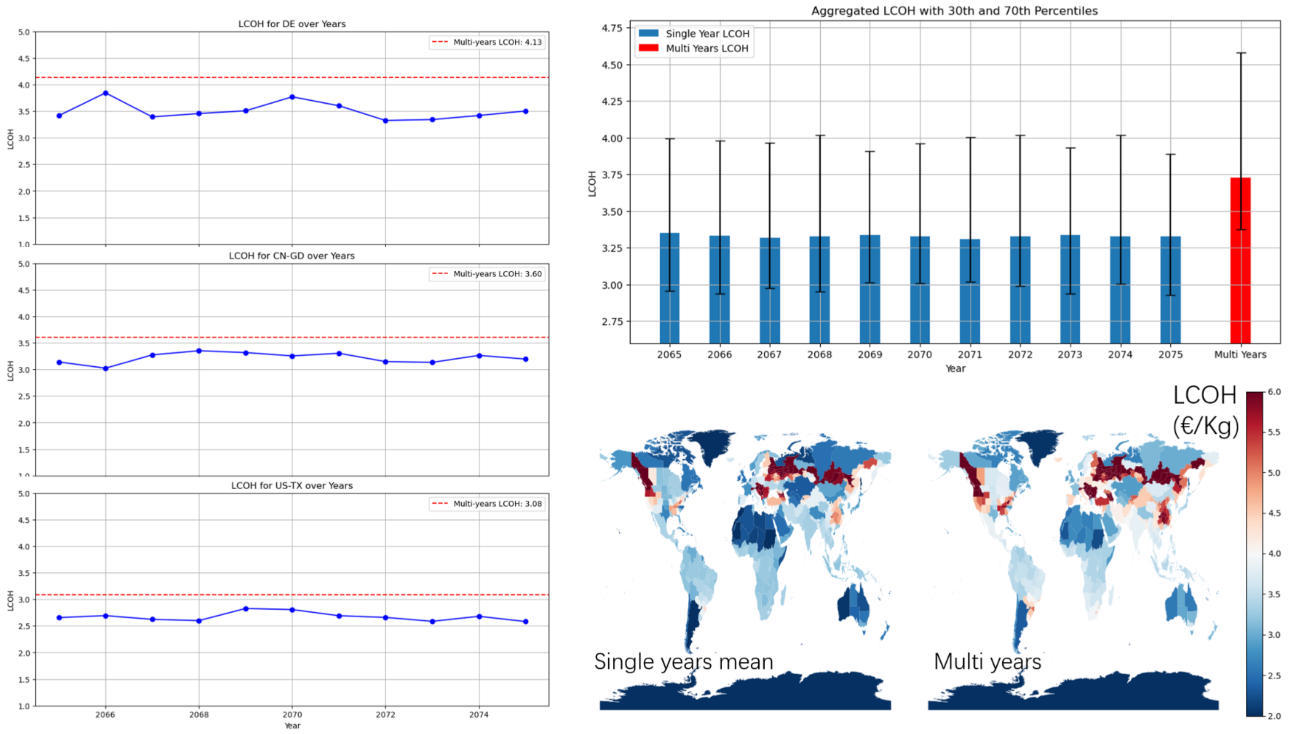

Figure S8: The LCOH under different yearly variability and multi-year reliable operation induced cost increase. The left column shows the yearly LCOH versus multi-year reliable operation LCOH, as marked in red dotted line for Germany (DE), Guangdong Province in China (CN-GD), and Texas state in the United States (US-TX). The right column, first row shows the global regions' mean LCOH under different yearly variability in blue bars with error bar indicating heterogeneity for regions' LCOH in 30% and 70% quantile, and red bar shows multi-year reliable operation induced cost increase. The maps in the second row show the spatial distribution of LCOH under different yearly variability and multi-year reliable operation-induced cost increase.

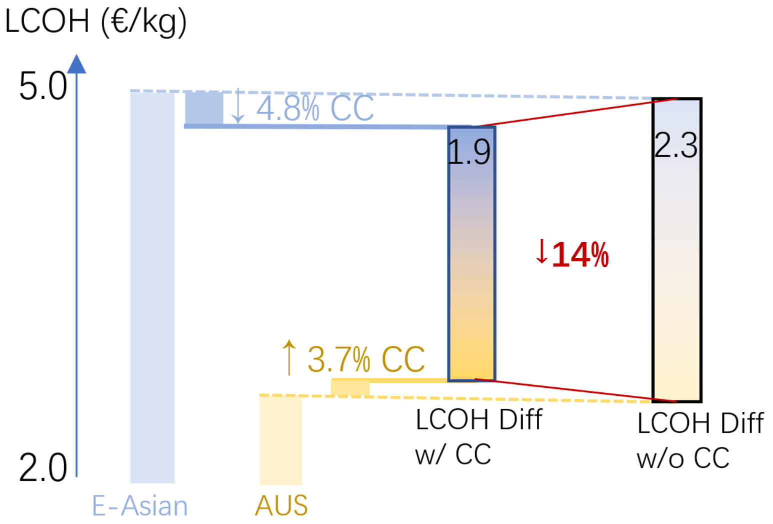

Figure S9: The cost difference between trade pair locations of exporting hydrogen from Australia (AUS) to East Asia (E-Asian) could be reduced due to climate change (CC). Larger change magnitude in cost differences between trade pairs could be observed than the magnitude of LCOH due to climate changes.

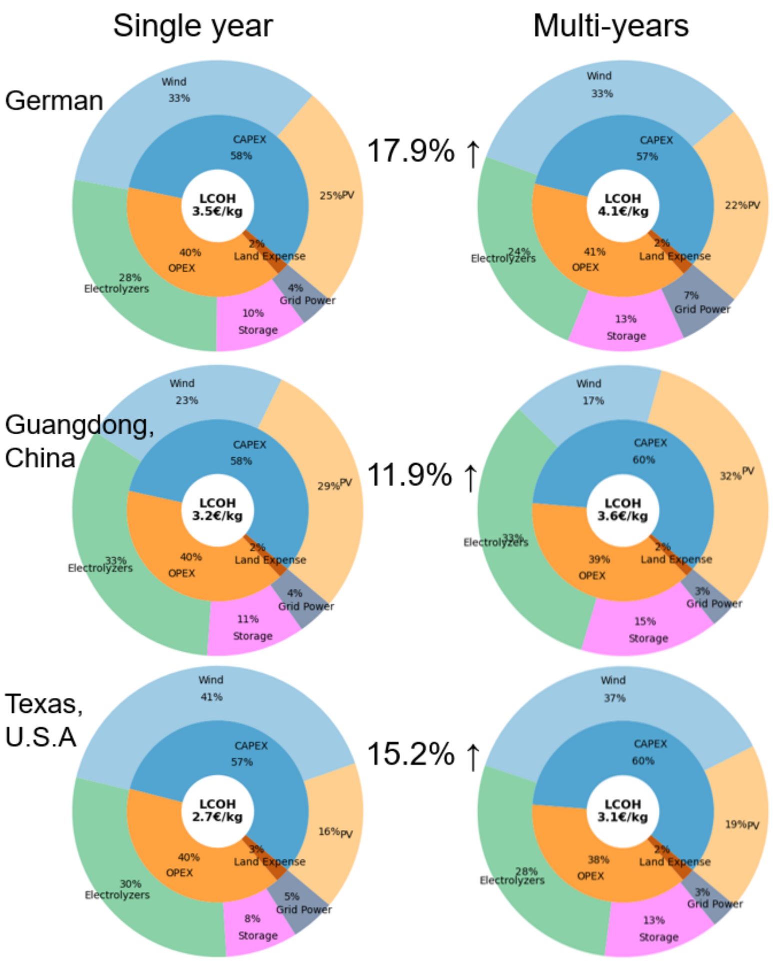

Figure S10: Cost breakdown for LCOH with different technologies investment, and CAPEX and OPEX changes, shown in pie chart, for a single year (left column) and multiple years (right). The three rows represent Germany, Guangdong Province in China, and Texas state in the United States.

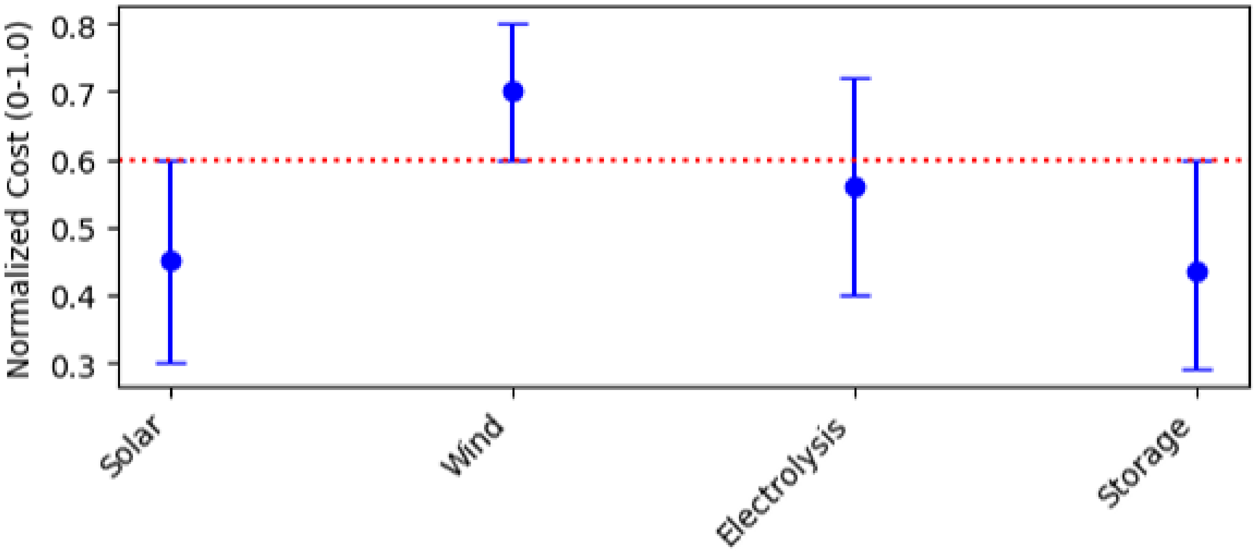

Figure S11: The cost reduction from contemporary to mid-century projection for different technologies, derived from[S10]. Red dotted line shows the overlap of all technologies of 60% cost reduction.

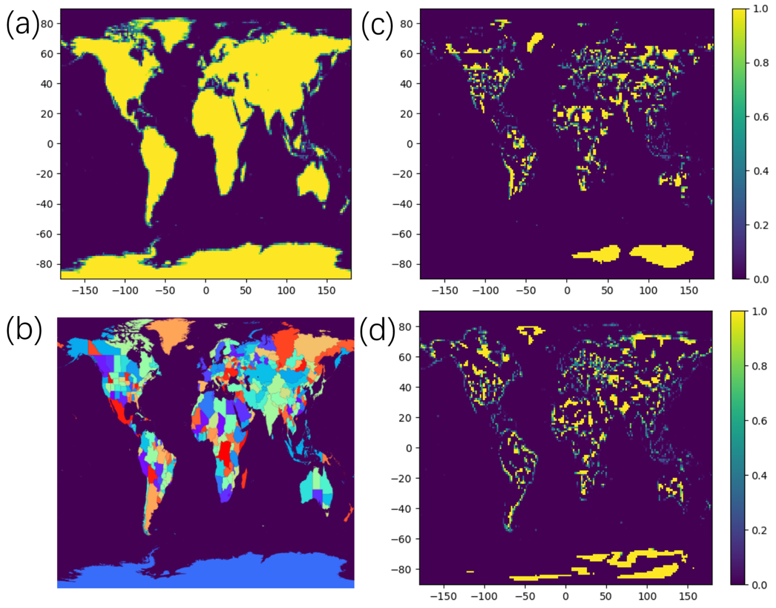

Figure S12: Spatial aggregation of different locations: (a) All global land, (b) Total 359 administrative regions, filtered to exclude regions with limited area. Colors are randomly selected to identify different region shapes. (c) Solar and (d) Wind selections, with the color bar showing the raster-based density weight for locations from 0 to 1, where 0 means no overlap with the selection and 1 means fully overlapped.

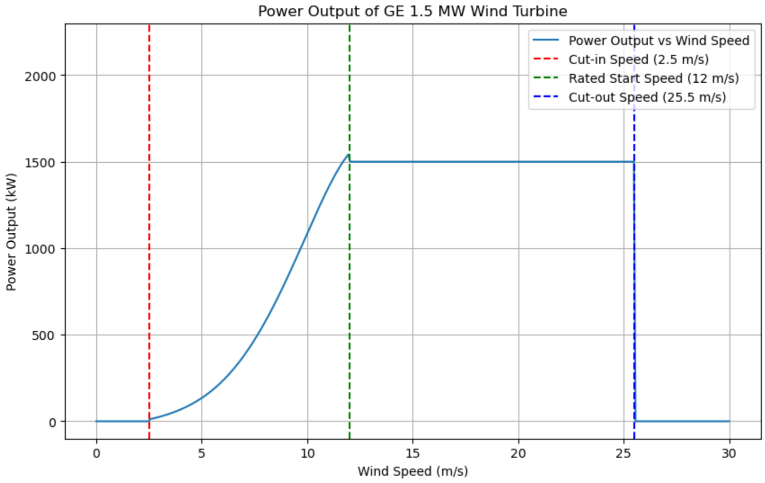

Figure S13: Wind profile for selected turbine to calculate wind capacity factor

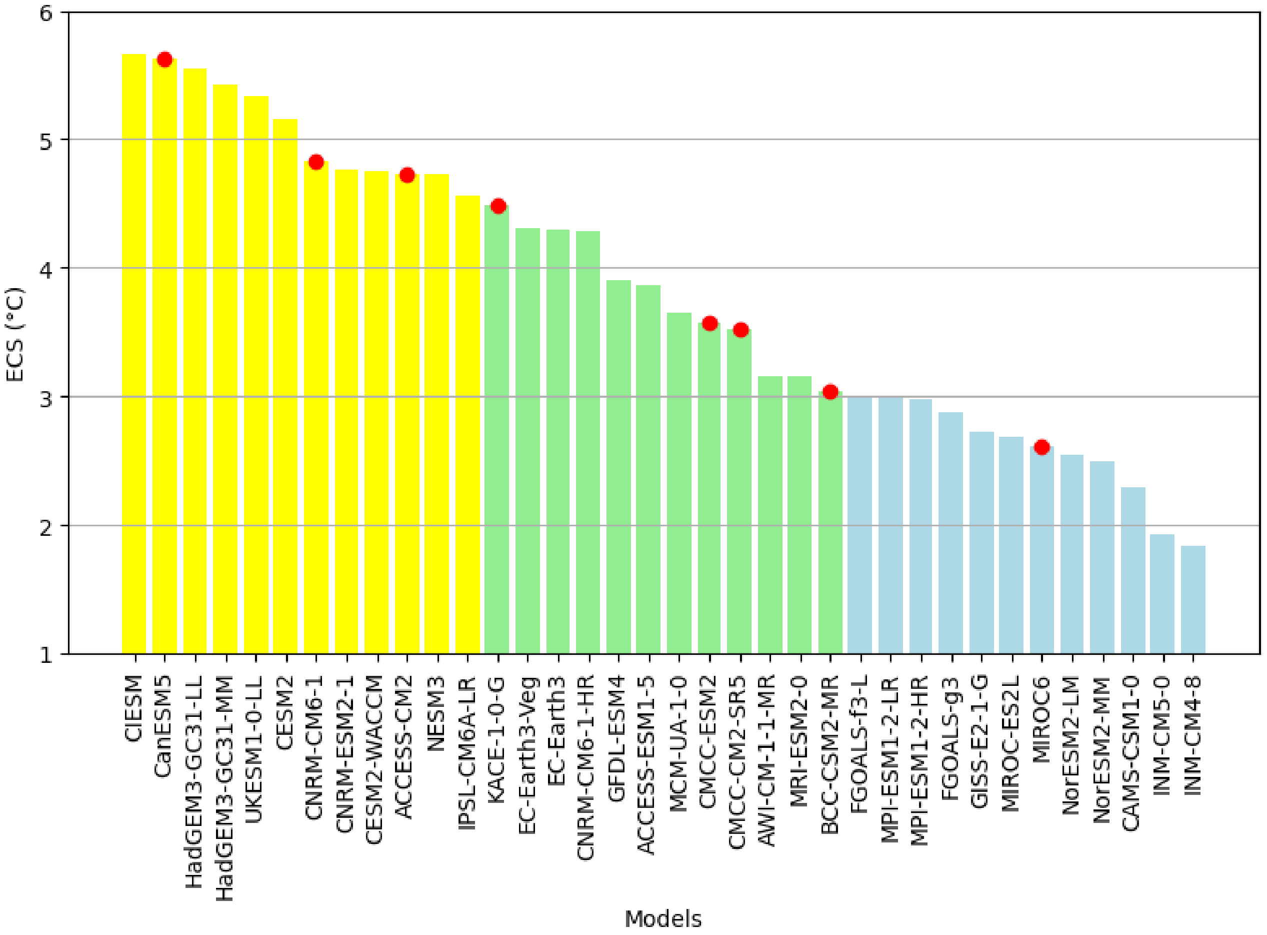

Figure S14: Equilibrium climate sensitivity (ECS) of selected 8 CMIP6 models (indicated by red dots on bar) in 36 CMIP6 models. The bar colors represent different ECS categories: yellow for high-ECS (4.51-6.00°C), green for medium-ECS (3.01-4.50°C), and blue for low-ECS (1.80-3.00°C). The data is derived from[S13,S14]

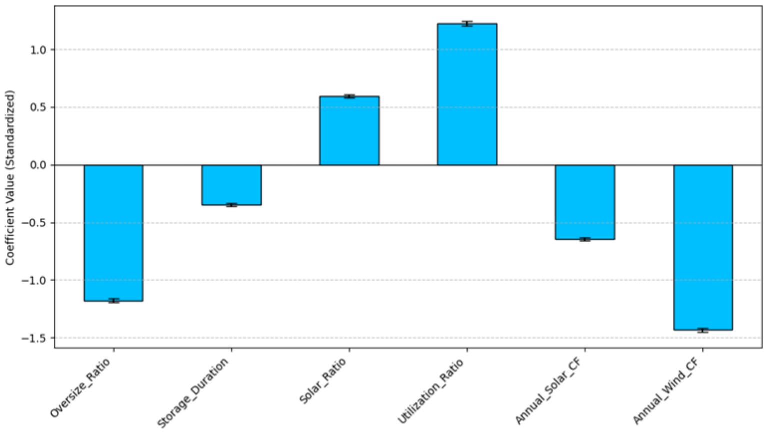

Figure S15: Standardized coefficients of the multivariate regression model explaining the absolute error between simplified and optimization-based LCOH models. The dependent variable is the LCOH error, and the model achieves an R^2 of 0.882. Bars represent the standardized ordinary least squares (OLS) coefficient for each predictor variable, indicating the magnitude and direction of its influence. Error bars depict the 95% confidence interval for each coefficient. All predictors are statistically significant (p ¡ 0.001).

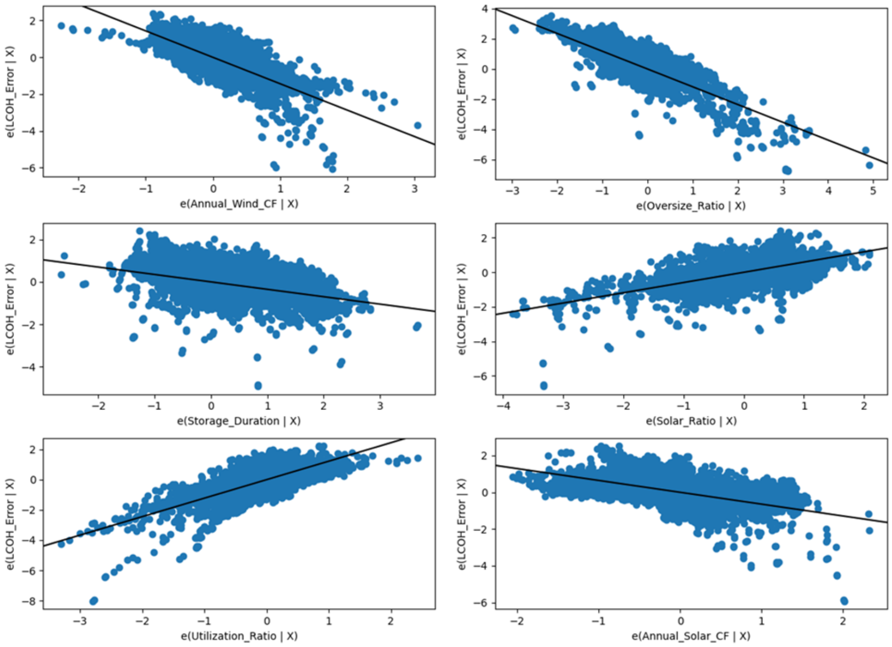

Figure S16: Partial regression plots for each predictor variable on the absolute LCOH error. Each subplot visualizes the relationship between the absolute LCOH error and a single predictor variable after accounting for the linear effects of all other variables in the model. The y-axis of each plot represents the residuals of the LCOH error regressed on all other predictors, while the x-axis represents the residuals of the selected predictor regressed on all other predictors. The slope of the fitted line in each subplot is mathematically identical to the coefficient for that variable in the full multivariate regression model (Supplementary Figure 17), visually confirming the unique contribution of each factor.

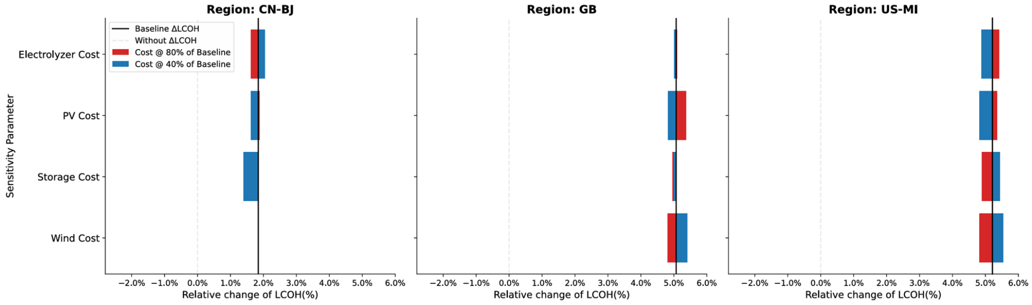

Figure S17: The impact of climate change on LCOH is robust to future technology cost reductions. Tornado plots show the sensitivity of the climate-induced relative change in LCOH (Δ LCOH) for three regions: Beijing (CN-BJ), Great Britain (GB), and Michigan (US-MI). The analysis individually varies the capital cost of each key technology (y-axis). The blue and red bars show the resulting Δ LCOH when a specific technology's cost is reduced to 40% and 80% of its contemporary value, respectively. The solid black vertical line in each plot represents the baseline Δ LCOH calculated in our main analysis. The minimal deviation of the bars from the baseline demonstrates that the projected percentage impact of climate change on LCOH is largely insensitive to a wide range of future, uneven technology cost reductions.

Supplementary References

1. Mingolla, S., Gabrielli, P., Manzotti, A., Robson, M. J., Rouwenhorst, K., Ciucci, F., Sansavini, G., Klemun, M. M., and Lu, Z. (2024). Effects of emissions caps on the costs and feasibility of low-carbon hydrogen in the European ammonia industry. Nature Communications *15*, 3753. doi:10.1038/s41467-024-48145-z.

2. Tonelli, D., Rosa, L., Gabrielli, P., Parente, A., and Contino, F. (2024). Cost-competitive decentralized ammonia fertilizer production can increase food security. Nature Food *5*, 469–479. doi:10.1038/s43016-024-00979-y.

3. Pan, G., Gu, W., Hu, Q., Wang, J., Teng, F., and Strbac, G. (2021). Cost and low-carbon competitiveness of electrolytic hydrogen in China. Energy & Environmental Science *14*, 4868–4881. doi:10.1039/D1EE01840J.

4. Song, S., Lin, H., Sherman, P., Yang, X., Nielsen, C. P., Chen, X., and McElroy, M. B. (2021). Production of hydrogen from offshore wind in China and cost-competitive supply to Japan. Nature Communications *12*, 6953. doi:10.1038/s41467-021-27214-7.

5. Bracci, J. M., Sherwin, E. D., Boness, N. L., and Brandt, A. R. (2023). A cost comparison of various hourly-reliable and net-zero hydrogen production pathways in the United States. Nature Communications *14*, 7391. doi:10.1038/s41467-023-43137-x.

6. Tonelli, D., Rosa, L., Gabrielli, P., Caldeira, K., Parente, A., and Contino, F. (2023). Global land and water limits to electrolytic hydrogen production using wind and solar resources. Nature Communications *14*, 5532. doi:10.1038/s41467-023-41107-x.

7. Terlouw, T., Bauer, C., McKenna, R., and Mazzotti, M. (2022). Large-scale hydrogen production via water electrolysis: A techno-economic and environmental assessment. Energy & Environmental Science *15*, 3583–3602. doi:10.1039/D2EE01023B.

8. de Kleijne, K., Huijbregts, M. A. J., Knobloch, F., van Zelm, R., Hilbers, J. P., de Coninck, H., and Hanssen, S. V. (2024). Worldwide greenhouse gas emissions of green hydrogen production and transport. Nature Energy 1–14. doi:10.1038/s41560-024-01563-1.

9. Brandt, J., Iversen, T., Eckert, C., Peterssen, F., Bensmann, B., Bensmann, A., Beer, M., Weyer, H., and Hanke-Rauschenbach, R. (2024). Cost and competitiveness of green hydrogen and the effects of the European Union regulatory framework. Nature Energy *9*, 703–713. doi:10.1038/s41560-024-01511-z.

10. Terlouw, T., Rosa, L., Bauer, C., and McKenna, R. (2024). Future hydrogen economies imply environmental trade-offs and a supply-demand mismatch. Nature Communications *15*, 7043. doi:10.1038/s41467-024-51251-7.

11. Karnauskas, K. B., Lundquist, J. K., and Zhang, L. (2018). Southward shift of the global wind energy resource under high carbon dioxide emissions. Nature Geoscience *11*, 38–43. doi:10.1038/s41561-017-0029-9.

12. Jerez, S., Tobin, I., Vautard, R., Montávez, J. P., López-Romero, J. M., Thais, F., Bartok, B., Christensen, O. B., Colette, A., Déqué, M., Nikulin, G., Kotlarski, S., van Meijgaard, E., Teichmann, C., and Wild, M. (2015). The impact of climate change on photovoltaic power generation in Europe. Nature Communications *6*, 10014. doi:10.1038/ncomms10014.

13. Lovato, T., Peano, D., Butenschön, M., Materia, S., Iovino, D., Scoccimarro, E., Fogli, P. G., Cherchi, A., Bellucci, A., Gualdi, S., Masina, S., and Navarra, A. (2022). CMIP6 Simulations With the CMCC Earth System Model (CMCC-ESM2). Journal of Advances in Modeling Earth Systems *14*, e2021MS002814. doi:10.1029/2021MS002814.

14. Scafetta, N. (2022). Advanced Testing of Low, Medium, and High ECS CMIP6 GCM Simulations Versus ERA5-T2m. Geophysical Research Letters *49*, e2022GL097716. doi:10.1029/2022GL097716.
